# A study on the effects of virtual high-altitude exposure on brain function activity in people with different severity of fear of heights

**DOI:** 10.3389/fpsyg.2026.1934322

**Published:** 2026-10-07

**Authors:** Chaolin Teng, Lin Cong, Qiumei Tian, Duoduo Hui, Meiqing Huang, Kaiwen Xiong, Weitao Dang, Yajing Hou, Jin Ma, Shan Cheng, Taihui Zhang, Chao Shen

**Affiliations:** 1 Department of Aerospace Medicine, Fourth Military Medical University, Xi'an, Shaanxi, China; 2 Department of Gastroenterology, The First Affiliated Hospital, Xi'an Medical University, Xi'an, Shaanxi, China; 3 The School of Computer Science and Engineering, Xi'an Technological University, Xi'an, Shaanxi, China

**Keywords:** electroencephalography microstate, fear of heights, microstate functional brain network, power ratio, weight phase lag index

## Abstract

**Introduction:**

The electroencephalography (EEG) microstate method has been extensively used to investigate brain function activity in patients with mental disorders. However, previous studies mainly focused on the analysis of basic microstate features. In this study, we aimed to explore brain electrical activities of individuals with different severity of fear of heights (FoH) during virtual high-altitude exposure using power spectrum and functional brain network based on microstate EEG.

**Methods:**

First, 69 male participants were included and classified into one of three degrees of FoH (i.e., no-FoH, mild or moderate FoH (m-FoH) and severe FoH (s-FoH)) according to their acrophobia questionnaire (AQ) scores. Then, 32-channel EEG signals were recorded from each subject during virtual high-altitude exposure. Subsequently, microstate EEG signals and corresponding microstate time windows for all subjects were obtained by the microstate analysis. Finally, the Welch method and weighted phase lag index were applied to these signals to compute microstate power spectrum features and brain network properties, and these measures were compared among three groups.

**Results:**

The results showed that the s-FoH group had significantly higher relative delta power than the no-FoH group for microstates B and F, and than the m-FoH group for microstates B, C and F. In constrast, the s-FoH group had lower relative alpha power than no-FoH group for microstates B, D and F, and than m-FoH group for microstates D and F. These differences were observed in multiple brain regions, mainly including the frontal and frontal-central areas. The s-FoH group also exhibited a significantly larger power ratio theta/alpha in the frontal-central region for microstates B, C, D and F compared to the no-FoH group, and in the central region for microstate D relative to the m-FoH group. Moreover, these features of microstate power spectrum with significant differences between different FoH groups significantly associated with the scores of AQ. For microstate functional brain network, prominently increased clustering coefficient and local efficiency were observed in both the m-FoH and s-FoH (i.e. FoH) groups for microstate F compared to the no-FoH group. It was also found that FoH individuals have significantly reduced robustness of microstate brain network F. Regarding degree distribution, the FoH groups exhibited power law distributions in microstate brain network A, whereas the no-FoH group displayed exponentially truncated power law patterns, which was opposite for microstate D.

**Discussion:**

In summary, our findings revealed the disrupted power spectrum and functional brain network in individuals with FoH during virtual high-altitude exposure from the perspective of microstate EEG, which may advance our understanding of the neuropathology of fear of heights and provide a novel electrophysiological tool for its clinical assessment. However, to control the influence of gender and age on fear responses and EEG dynamics, only young male participants in their early twenties were recruited. Therefore, the present findings should be interpreted with caution, and their generalizability to female populations and older adults remains to be verified.

## 1 Introduction

Fear of heights (FoH) is one of the most prevalent specific phobias with a lifetime prevalence of ~3%−6% (Huppert et al., 2020; Francová et al., 2025), which is characterized by intense and irrational fear associated with height-related stimuli such as tall buildings, ladders, cliffs, or sky wheels. To avoid this distressing experience, individuals with FoH often avoid such trigger situations, leading to restricted aspects of their daily lives and career choices (Brandt and Huppert, 2014). As a psychiatric disorder, FoH has an increasing incidence as more people are exposed to high-altitude environments, gradually becoming a public health concern. However, many individuals fail to recognize it as a clinical condition (Hong et al., 2017) and therefore miss the optimal window for seeking hospital treatment, resulting in progressively worsening symptoms.

Previous research based on resting state functional magnetic resonance imaging (rs-fMRI) has revealed disrupted brain function in orbitofrontal cortex, medial prefrontal cortex, and visual regions in patients with FoH (Hang et al., 2022). Another rs-fMRI study found that FoH patients exhibited local abnormalities in spontaneous neural activity and functional connectivity (FC) in the visual cortex, cerebellum and orbitofrontal cortex (Guo et al., 2023). Due to the limitations of fMRI equipment, these studies could only analyze the brain function activities of individuals with FoH during the resting state. Fortunately, virtual reality (VR) technology can simulate high-altitude scenarios, which has been found to be at least as effective as real-world exposure (Francová et al., 2025; Kvapil Varšová and Jurík, 2024). On the other hand, electroencephalogram (EEG) recorded by electrodes placed on the human scalp directly reflects important information of electrical activity linked to the central nervous system, which is widely used in the field of brain science research (Shen et al., 2023; Chetty et al., 2024; Gordillo et al., 2023). Therefore, the combination of VR and EEG enables the exploration of brain electrical activity during virtual high-altitude-induced fear, which could further enhance our understanding of the neural mechanisms underlying fear of heights. For example, some investigators have used this approach to identify the severity of FoH (Wang et al., 2019; Wang Q. et al., 2021). The results indicated that there were significant differences in the brain network properties among individuals with different levels of FoH and characteristics extracted from brain network were verified to be able to effectively assess the degree of FoH. In conclusion, the combined use of VR and EEG techniques offers unique advantages in investigating the brain function activity of different FoH individuals during the virtual high-altitude exposure. However, up to now, research on FoH remains very limited.

EEG microstate, first proposed by Lehmann et al., has been utilized to investigate the synchronized and stable scalp potential patterns at the millisecond time scale (Lehmann et al., 1987), reflecting the instantaneous global electrical activity of the brain. Generally, there are some representative microstates appearing in awake EEG and each type of microstate commonly maintains stable for approximately tens of milliseconds before rapidly shifting to another microstate (Koenig et al., 2002; Khanna et al., 2014; Milz et al., 2016; Qi et al., 2024). Growing evidence showed that the temporal dynamics of the microstates were abnormally disrupted in neurological and psychiatric disorders, including major depression (Murphy et al., 2020; Zhao et al., 2024), schizophrenia (Niu et al., 2022; Yan et al., 2023) and obsessive-compulsive disorder (Thirioux et al., 2023). Furthermore, our previous study compared basic EEG microstate features among different FoH groups under virtual high-altitude condition and identified significant differences in microstate F characteristic parameters between no-FoH and FoH groups (Teng et al., 2024). Therefore, these findings suggest that the EEG microstate analysis is a promising and effective spatio-temporal method that could serve as an auxiliary tool for the diagnosis of clinical mental disorders.

EEG microstates have been shown to be closely associated with intrinsic functional brain networks that remain active during the resting state, with different microstates reflecting distinct patterns of neural resource allocation and reorganization (Britz et al., 2010; Michel and Koenig, 2018; Yuan et al., 2012; Custo et al., 2017). For instance, Michel and Koenig reported in a review article that microstate A was related with negative blood oxygen level-dependent (BOLD) activation and corresponded to the auditory network; microstate B was correlated with negative BOLD activation and corresponded to the visual network; microstate C was associated with positive BOLD activation and corresponded to the saliency network; microstate D was related with negative BOLD activation and corresponded to the attention network (Michel and Koenig, 2018). So, the exploration of microstate functional brain networks could provide novel insights into the underlying pathology of these disorders (Hao et al., 2022; Chu et al., 2023; Zhang et al., 2024). Specifically, researchers first applied microstate analysis to EEG signals to generate microstate sequences, then extracted microstate time windows corresponding to each microstate label, and finally constructed each type of microstate functional brain network by applying functional connectivity approaches to these time windows. However, to the best of our knowledge, no studies have yet investigated microstate functional brain networks in fear of heights. Based on our previous EEG microstate study on FoH (Teng et al., 2024), the present study aimed to further investigate brain function in individuals with different FoH levels by analyzing microstate power spectrum and microstate functional brain networks, thereby providing sensitive electrophysiological indicators for assessing FoH severity and understanding its underlying neural mechanisms. Besides, previous neurophysiological studies have confirmed that both gender and age significantly influence fear responses, brain electrical oscillations and functional connectivity patterns (McLean and Anderson, 2009; Van Ruitenbeek et al., 2023; Lallement and Lemaire, 2025). To eliminate these confounding factors in EEG and emotion research, the current study exclusively recruited young male participants aged in their early 20s. Restricting enrollment to a homogeneous young male sample effectively reduces individual heterogeneity caused by demographic differences and improves internal validity. Nevertheless, such homogeneous sample inevitably limits external validity, and future studies with mixed genders and wider age ranges are required to verify the generalizability of the findings in the study.

## 2 Materials and methods

### 2.1 Participants

The study included 69 male participants, which were derived from our previous study (Teng et al., 2024). The total sample size of *N* = 69 was therefore based on convenience and availability rather than determined in advance by power analysis. These participants were divided into three groups according to the scores of acrophobia questionnaire (AQ): the no-FoH with AQ-anxiety ≤ 20 and AQ-avoidance ≤ 6 (*N* = 20, mean age = 22.10 ± 1.55 years), severe FoH (s-FoH) with AQ-anxiety ≥ 40 and AQ-avoidance ≥ 12 (*N* = 24, mean age = 22.08 ± 2.45 years) groups, and the remaining participants belonged to mild or moderate FoH (m-FoH; *N* = 25, mean age = 23.16 ± 2.76 years). Each subject reported no vestibular and balance deficits, other neurological or orthopedic disorders. The informed consent forms were provided by all the participants before participating in the experiment. Besides, the ethical consent of this study was obtained through the Ethics Committee of Air Force Medical University. Please refer to our previous study for more details on demographic information (Teng et al., 2024).

### 2.2 Virtual high-altitude exposure experiment

The construction of virtual environment was realized by Unity 3D software (Unity Technologies Inc, San Francisco, USA), and the virtual high-altitude scenario simulation was implemented by An HTC VIVE Pro Eye, which was used to induce fear of heights. The virtual high-altitude exposure experiment was shown in Figure 1. We chose the maximal 260 meters height in the virtual environment to guarantee that each individual was under the state of fear of heights to the greatest extent. After understanding the experimental procedures, the participant was instructed to wear the HTC VIVE Pro Eye and stood the end of a transparent plank hanging at a height of 260 meters from the edge of the roof in the virtual world. In the real world, the virtual plank was mapped from a physical plank, which had 4.8 meters length and 0.5 meters width. It should be noted that the virtual height did not changed over the course of the experiment. When the subject stood on the plank, the springs installed beneath the plank will vibrate slightly due to gravity, which can further enhance the sense of fear. Then, the experimenter asked participant to look down by lowering his head slightly during the virtual high-altitude exposure. Besides, the subjects were also required to keep their bodies as still as possible during the entire experimental process, so very few motion artifacts were generated. In this way, the collected EEG signals were minimally affected by motion artifacts.

**Figure 1:**
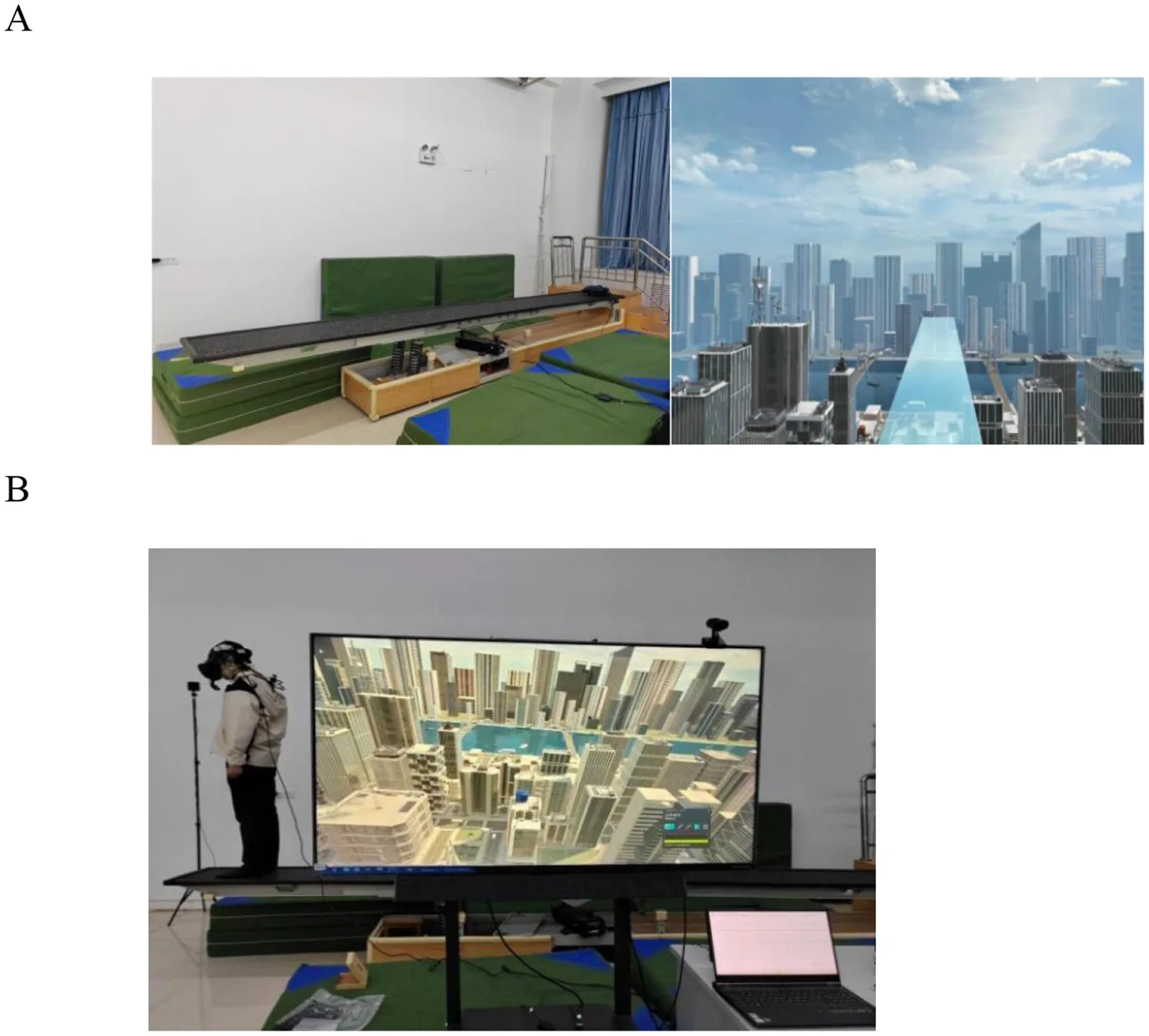
Virtual high-altitude exposure experiment. **(A)** A physical plank in the real world and a transparent plank mapped by an HTC VIVE Pro Eye in the virtual world. **(B)** A subject was exposed to the virtual high-altitude scene.

### 2.3 EEG signals acquisition and pre-processing

The continuous EEG signals were recorded for 3 min with a sampling frequency of 500 Hz by a 32-channel Quik-Cap of Brain Products with portable LiveAmp amplifier (Brain Products, German) when the participants looked down straightly from the virtual high-altitude. Each electrode was placed in accordance with the standard international 10–20 system. The placement of ground electrode was at the middle of the Fp1 and Fp2 electrodes. The reference electrode was positioned at between the Fz and Cz electrodes. Subsequently, the EEG data were pre-processed by the version of MATLAB R2021a software (The MathWorks Inc., USA) equipped with the EEGLAB toolbox (Delorme and Makeig, 2004), which comprised the processes of reference transformation to the average reference, down-sampling to 256 Hz, a band-pass filtering of 2-20 Hz by a zero-phase finite impulse response (FIR) filter to minimize the effects of high-frequency noise and low-frequency artifacts from the VR headset-related electromagnetic equipment, physiological signal artifacts removal by independent component analysis (ICA). In addition to ICA for removing artifacts, the initial 10 s and final 10 s of EEG signals were discarded to eliminate transient artifacts, leaving a middle valid duration of 2 min 40 s data for each subject. For the retained EEG signals, the inspected residual artifacts including the vibration of the physical plank, head movement and body motion were manually and directly removed. After artifact removal, we randomly selected a continuous 2-min artifact-free EEG data from each subject for further analysis. Among them, the ICA method was employed to mainly reject the artifactual components originating from physiological signals, which include electrooculography (EOG), electrocardiography (ECG) and electromyography (EMG). Compared with the EEG signal, the waveforms of these artifactual components and their energy distribution on the scalp are more regular and easier to distinguish. For instance, the amplitude of EOG signal is much higher than that of EEG, and the energy topographic distribution of the EOG component is mainly located at the prefrontal region. In the study, the number of ICA components excluded in different FoH groups was 2.45 ± 1.00 (no-FoH), 3.12 ± 1.39 (m-FoH), and 3.25 ± 1.68 (s-FoH), respectively. The statistical result showed that there was no significant difference among these three sets of data (Kruskal–Wallis test, *H* = 2.316, *p* = 0.314). Besides, it is noted that there is no standard for the sequences of the EEG pre-processing process at present. Some studies performed reference conversion first and then ICA transformation (Liu et al., 2020; Zhao et al., 2022), while others performed ICA transformation first and then reference conversion (Langer et al., 2013; Hasanzadeh et al., 2020). And, the results of these researches indicated that both pre-processing ways were effective. Therefore, the study selected one of the methods, that is the former one, for processing the collected EEG signals.

### 2.4 Brain function activity analysis

The band-pass filtered signal ranging from 2 to 20 Hz was used in our previous microstate analysis study (Teng et al., 2024). The selection of this frequency range was based on a large body of microstate studies (Seitzman et al., 2017; Schiller et al., 2019; Pan et al., 2020; Zhao et al., 2022; Li et al., 2022), which has been shown to yield the most stable and reproducible microstate topographies. For microstate analysis, briefly, the EEG signals at global field power (GFP) peak points as representative maps were first selected from all subjects. Second, the modified *k*-means method (regardless of signal polarity) was employed on the selected potential maps to obtain microstate templates and the optimal number of microstates was determined by the global explained variance (GEV) and cross-validation (CV) criteria (five in the previous study). After the derivation of the microstate templates (labeled A, B, C, D and F), the obtained microstate templates were fitted back to all original maps for each subject. By calculating the global map dissimilarity (GMD) between each instantaneous EEG topographic map and all template maps, the EEG map was labeled as one of the five microstate templates with the minimal GMD value. During the back-fitting procedure, the smoothing parameters were set as temporal smoothing width parameter assigned the value of 3, temporal smoothing weight parameter assigned the value of 5, and the minimum segment duration parameter assigned the value of 30 ms. The mean GEV values for the no-FoH, m-FoH and s-FoH groups across five microstate templates were 0.61, 0.62 and 0.63, respectively. In addition to the four canonical microstates (A-D), a non-canonical microstate F was identified in our previous study (Teng et al., 2024). As previously interpreted, microstate F could be associated with the brain state alterations caused by fear elicited by virtual high-altitude exposure, which plays an important role in the neural regulation of virtual high-altitude-induced brain states. Based on our previous research, we further analyzed the brain function activity in individuals with different severity of FoH under the condition of virtual high-altitude exposure from the perspective of microstate EEG. Our previous study have conducted the microstate method on pre-processed EEG signals, which generated five types of microstate classes. So the microstate sequence of each subject was acquired. Thus, the same type of microstate time windows were concatenated to generate microstate EEG signals for computing power spectrum features for the power spectrum analysis on the one hand; according to labels of each microstate in the microstate sequence, each piece of time window data belonging to the same type of microstate was extracted and applied to calculate functional connectivity matrices for the analysis of microstate brain network on the one hand. The flow diagram of brain function activity analysis based on microstate EEG is shown in Figure 2, which consists of microstate power spectrum analysis and microstate functional brain network analysis.

**Figure 2:**
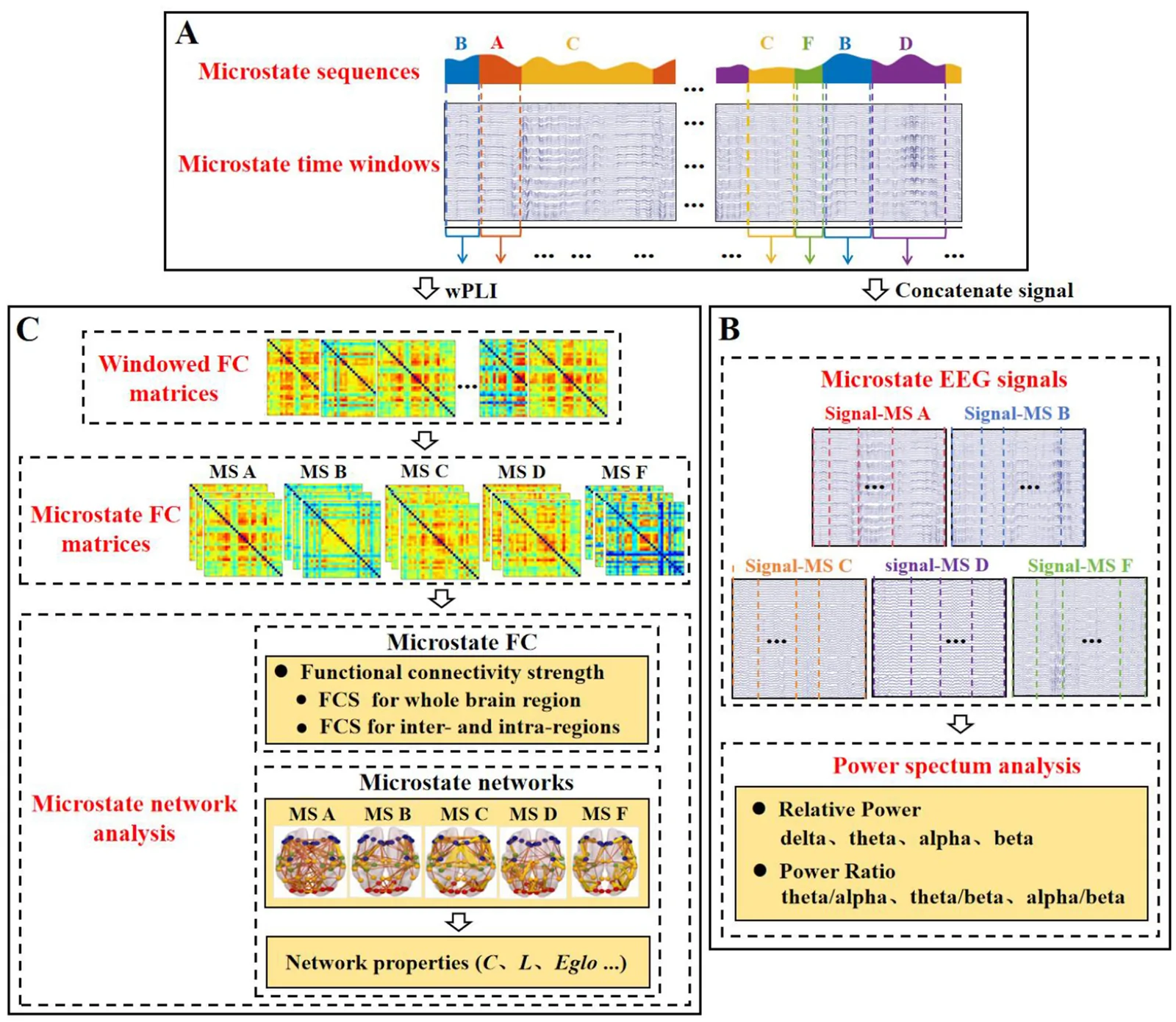
The flow chart of brain function activity analysis based on microstate EEG. **(A)** Extraction for each piece of microstate-window-based EEG signal. **(B)** The process of microstate power spectrum analysis. **(C)** The process of microstate functional brain network analysis. wPLI, weighted phase lagged index; FC, functional connectivity; FCS, functional connectivity strength; MS, microstate.

#### 2.4.1 Microstate power spectrum analysis

In the current study, all temporally discontinuous EEG segments belonging to the same type of microstate were directly concatenated to obtain microstate EEG signals. Then, the power-spectrum-related features, that is the power of four basic EEG rhythms (i.e., delta: 2–4 Hz; theta: 4–7 Hz; alpha: 8–12 Hz; beta: 13–20 Hz), were computed from each type of microstate EEG signals by applying the Welch method with a 2-s, 50% overlap sliding Hanning window. For these boundaries between originally non-contiguous EEG segments, we did not performed any additional processing (e.g., tapering, padding or another procedure). After obtaining the absolute power values of the four frequency bands for each subject, to reduce inter-individual differences in EEG power we calculated the relative power of each frequency band by dividing its absolute power by the total absolute power of the four frequency bands within the same subject; another power characteristic for microstate EEG signals was the ratio of the power of different basic EEG rhythms. Since different power ratios of EEG rhythms were regarded as the effective indicators for identifying different brain states such as (theta + alpha)/beta for fatigue (Hu and Lodewijks, 2020), alpha/beta for visual memory induced by cognitive tasks (Tempel et al., 2020), and theta/alpha and theta/beta for neurocognitive disorders (Cheung et al., 2018), the latter three power ratios, namely theta/alpha, theta/beta and alpha/beta, were selected in the study to assess the degree of FoH due to the initial exploration.

#### 2.4.2 Microstate functional brain network analysis

For microstate functional brain network analysis, firstly, the weight phase lag index (wPLI) approach was employed to calculate the strength of FC for every pair of EEG channels in microstate time window signal. The wPLI method is a phase synchronization measure that is more robust for volume conduction problems (Vinck et al., 2011). Given two signals *X* and *Y*, its mathematical expression is defined as follows:


$$\begin{array}{l} wPLI_{XY}=\frac{N^{-1}\sum_{i=1}^{N}|imag\left(S_{XY}\right)|sign\left(imag\left(S_{XY}\right)\right)}{N^{-1}\sum_{i=1}^{N}|imag\left(S_{XY}\right)|} \end{array}$$ (1)

where *N* represents the total number of sample points for the signal *X* (*Y*), *imag*(*S*_*XY*_) denotes the imaginary part of the cross-spectral density of *X* and *Y*, and *sign*(·) is the symbolic function. For cross-spectral estimation, the Hilbert transform was employed to derive the analytic signal for each signal in the study. Specifically, the analytic signals *H*(*X*) and *H*(*Y*) of *X* and *Y* were first obtained using the Hilbert transform respectively. Then, the cross-spectral density was computed as by *S*_*XY*_ = *H*(*X*)·H^*^(*Y*), where ^*^ denotes the complex conjugate. Subsequently, the wPLI was derived from the imaginary component of this cross-spectrum. The wPLI has a value range of 0 to 1, and the greater the value, the higher the phase synchronization. Besides, microstate EEG signals with 2–20 Hz were used for wPLI calculation, consistent with the frequency range used in the microstate analysis.

##### 2.4.2.1 Functional connectivity strength (FCS): FCS for the whole brain region and the seven sub-regions

As microstates are intrinsically transient, a minimum duration of accepted microstate segment was set to 70 ms in the study. This threshold was chosen as a compromise between retaining a sufficient number of microstate windows for statistical power and maintaining estimation stability. Segments of microstate window shorter than 70 ms were excluded from all subsequent wPLI calculations. By using wPLI, FC matrices were separately computed for every valid microstate segment. Then, for each participant and each class of microstate, these segment-level FC matrices were averaged to obtain the final class-specific FC. Subsequently, the functional connectivity strength (FCS) of matrices were estimated for both the whole brain region and four seven sub-regions for the comparative analysis of different FoH groups. The seven sub-regions included the left-upper (Fp1, F7, F3, FT9, FC5, FC1), right-upper (Fp2, F4, F8, FC2, FC6, FT10), left-lower (CP5, CP1, P7, P3, O1), right-lower (CP2, CP6, P4, P8, O2), left-middle (C3, T7), right-middle (C4, T8) and midline (Fz, Cz, Pz, Oz) brain regions [as for the location of brain regions, please refer to Figure 2 in Teng et al. (2024)].

##### 2.4.2.2 Brain network topology: basic network properties, degree distribution, hub nodes and robustness.

Before brain network construction, to minimize the influence of noisy and spurious connections and improve the computational efficiency (Wang et al., 2016), each FC matrix was thresholded into a binarized matrix with a fixed sparsity value (defined as the ratio of the total number of edges to the maximum possible number of edges in a network). Currently, there are no standard recommendations for sparsity selection, and different sparsity ranges have been selected for brain network analysis in previous studies [e.g., 10%−46% (Tian et al., 2011), 65%−95% (Langer et al., 2013), 5%−55% (Li et al., 2015), 1%−10% (Hasanzadeh et al., 2020)]. So, in the study, a wide set of sparsity ranging from 10% to 50% at an interval of 0.5% was chosen for the computation of network topological attributes. Then, the graph theory analysis method was applied to explore important information about brain network topology. In graph theory, a network is defined as a set of nodes representing channels of the study and edges representing connections between channels.

There are a wide variety of metrics for quantitatively measuring the topological properties of a network, and different metrics can reflect the characteristics of the network from different perspectives. Referring to numerous previous studies on normal brain network and brain networks of patients with mental disorders (Liu et al., 2020; Hasanzadeh et al., 2020; Wang et al., 2016; Tian et al., 2011; Li et al., 2015; Wang Q. et al., 2021), the study selected representative and commonly used network properties to characterize the topology of brain networks in people with different severity of FoH. These basic network properties including characteristic path length (*L*), clustering coefficient (*C*), global efficiency (*E*_*glo*_), local efficiency (*E*_*loc*_), normalized clustering coefficientγ = *C*/*C*_*rand*_, normalized characteristic path length λ = *L*/*L*_*rand*_, normalized global efficiency (normalized$E_{glo}=E_{glo}/E_{glo}^{rand}$) and normalized local efficiency (normalized $E_{loc}=E_{loc}/E_{loc}^{rand}$) were computed for each type of microstate brain network for all subjects, where *C*_*rand*_, *L*_*rand*_, $E_{glo}^{rand}$and $E_{loc}^{rand}$are the means of corresponding measures produced by the matched 100 random networks preserving the same nodes, edges, and degree distribution in the original network (Maslov and Sneppen, 2002). The topological metrics and the construction of random networks above were calculated by using a freely available Brain Connectivity Toolbox (Rubinov and Sporns, 2010) on the platform of MATLAB R2021a software. Since a variety of network metrics were calculated by a set of graphs generated by the set of sparsity values, a functional curve can be mathematically obtained: the x-axis denotes the sparsity and y-axis denotes the network metric value. In order to determine whether there were any group differences of these curves, the Functional Data Analysis as a common machine learning method was employed for characterizing and comparing graph curves between different FoH groups (for detailed information, please see Bassett et al., 2012; Vico Fallani et al., 2014).

The node degree represents the number of edges directly linked to the node, which is an important characteristic of the network topology. The degrees of all nodes in the network constitute the degree distribution that is a crucial marker of network development and robustness. Generally, the degree distribution of the brain network has the following three forms: a power law, *P*(*k*) ~ *k*^α^−1; an exponential, *P*(*k*) ~ *e*^*ak*^; and an exponentially truncated power law, *P*(*k*) ~ *k*^α^−1e^k/*kc*^. Where *k* is the degree of the node, α represents the power law exponent, and *kc* denotes the exponential truncation. In this study, these three distribution functions were applied to fit the probability of node degree and the degree of fitting was evaluated using the coefficient of determination *R*^2^, where the closer the value *R*^2^ is to 1, the better the goodness-of-fit (Gong et al., 2009). In order to explore the distributed characteristic of nodes in the microstate brain network, the sparsity of 23.5% was selected to compute the degree distribution here for the comparison analysis in the study, which was based on the fact that the average degree of the nodes can be guaranteed to be equal to 2^*^log(*N*) (log represents the logarithmic function with the base of the natural constant e, and *N* represents the total number of network nodes; *N* = 30 in the study) under the condition of this sparsity (Wang S. et al., 2021; Achard and Bullmore, 2007). The selected sparsity not only satisfied the minimum sparsity requirement, but also controlled the false connections in the network to the greatest extent. Therefore, the subsequent network analysis was based on this sparsity.

The hub node refers to the node that plays a key role in the network, which is equivalent to “connection hub.” Typically, the regional hubs are connected to more nodes in the network relative to non-hubs, so the degree of a hub node is relatively large. To reduce the differences of node degrees between individuals, the mean degree of a node were divided by the mean degree of all nodes for all subjects to obtain the normalized degree of a node. In the present study, a node was identified as a hub if the degree of the node was greater than the sum of the mean and standard deviation of all node degrees.

Network robustness is characterized by the tolerance level of a complex network against random failures or targeted attacks, reflecting the stability characteristics of the network. In the study, the robustness of microstate functional brain network in individuals with different degrees of FoH was investigated through the nodes or edges removal. For random failure of a network, a node was randomly selected from all nodes and was removed (including the edges connected to the node). After removing the node, the size of largest cluster and the global efficiency of the network were calculated. Then we chose another node at random in the remaining nodes and recalculated these two indices in the remaining network. The process was repeated until the size of largest cluster was 1. Then a series of the largest cluster values were obtained for each subject. Since the maximum connected clusters varied across subjects before the nodes the relative size of largest cluster was calculated by dividing the maximum value among all the largest cluster values of the subject by the corresponding size of largest cluster to reduce individual differences for robustness assessment. The same procedure was also applied to the targeted attack, but the nodes were eliminated in the descended order of all node degrees. Unlike the targeted attack, to get reliable and convincing results the whole process of the random failure was simulated 1,000 times, and the performance of network robustness was evaluated based on the average of corresponding indices.

### 2.5 Statistical analysis

In this study, the statistical analysis was implemented by IBM SPSS Statistics 27 software (IBM Inc., New York, USA). If the data of the no-FoH, m-FoH and s-FoH all meet the normal distribution condition, one-way analysis of variance (ANOVA) method was performed to test the prominent changes then pairwise comparisons between groups were checked using the simple *post-hoc* test; other using non-parametric Kruskal–Wallis one-way ANOVA test for multiple independent samples. Multiple comparisons across groups were corrected by the Bonferroni method. Furthermore, if there was a significant difference in relative power or power ratio characteristic of each type of microstate EEG signal across electrodes between any two groups with different levels of fear of heights, the simple linear regression model was employed to further investigate their relationships with the scores of AQ-anxiety and AQ-avoidance, respectively. Specifically, for each microstate EEG power feature, the Bonferroni correction was performed when correlation analyses involved three or more brain regions. Both raw and corrected *p* values were reported in the tables of the subsequent correlation analysis results. When only one or two brain regions were analyzed, a limited number of statistical tests were conducted, so no multiple comparison correction was performed and only raw *p* values were presented. In addition, all analyses were conducted at *p* < 0.05 with a statistical significance.

## 3 Results

### 3.1 Comparisons of microstate EEG relative power between different FoH groups

The distributions of brain topographic maps for average relative power of delta, theta, alpha and beta bands in different FoH groups for EEG signals of microstate F are shown in Figure 3 (see Supplementary Figures 1–4 for brain topographies of relative power for EEG signals of microstates A–D). It can be seen from the figure that both the m-FoH and s-FoH (i.e. FoH) groups exhibited comparatively higher relative power in delta band but lower relative power in alpha band in the frontal region in comparison with the no-FoH group. Besides, reduced relative delta power in central-parietal and parietal areas for microstate F was observed in the no-FoH group.

**Figure 3:**
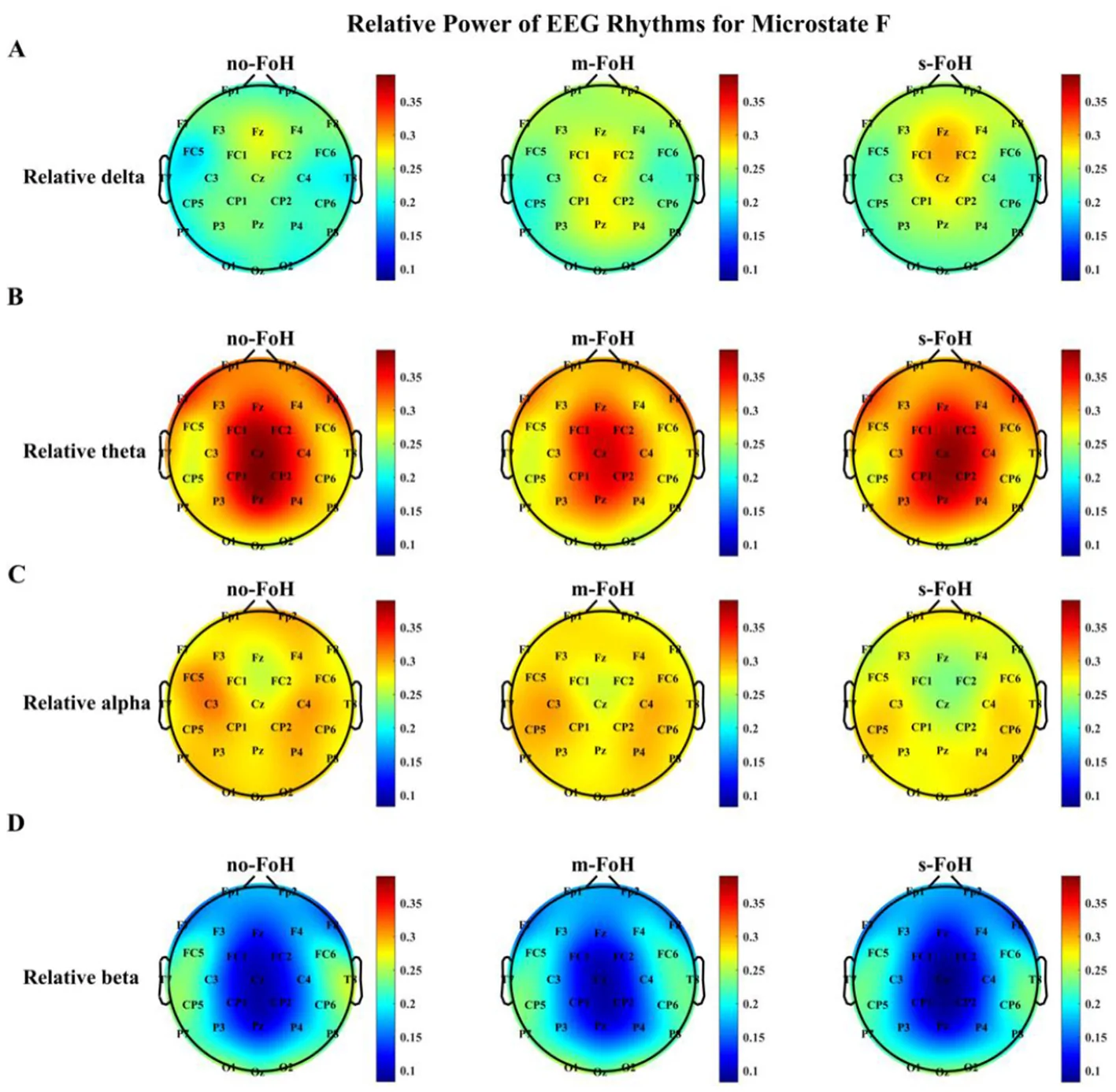
Distributions of brain topographic maps for average relative power of basic EEG rhythms in different FoH groups for microstate F. Relative **(A)** delta, **(B)** theta, **(C)** alpha, and **(D)** beta power. Each row of each subgraph represents the average relative power results of the no-FoH, m-FoH and s-FoH groups based on EEG signals of microstate F. FoH, fear of heights; the no-FoH, m-FoH and s-FoH denote no, mild or moderate and severe fear of heights, respectively.

Table 1 provides the statistical results of EEG channels with significant differences in relative power comparisons of people with different levels of FoH for microstate F. It can be seen from the table that compared to the no-FoH group, significantly greater relative delta power was observed in the FoH groups in multiple brain regions including frontal (Fp2), frontal-central (FC1 and FC5), central-parietal (CP2) and parietal (P4 and Pz) regions. The s-FoH group also had remarkably decreased relative alpha and beta power in Fz than the m-FoH group.

**Table 1:** Statistical results of EEG channels with significant differences in relative power of EEG rhythms between different FoH groups for microstate F.

Microstate type	Frequency band	Channel (F,η^2^,*p*)	Group			*pcorr*		
			no-FoH mean (std)	m-FoH mean (std)	s-FoH mean (std)	no-FoH vs. m-FoH	no-FoH vs. s-FoH	m-FoH vs. s-FoH
F	Delta	Fp2 (8.51,0.14,0.001)	0.21 (0.03)	0.26 (0.06)	0.25 (0.06)	**0.003**	**0.021**	0.859
		Fz (4.51,0.12,0.017)	0.27 (0.03)	0.26 (0.06)	0.29 (0.04)	0.784	0.068	**0.027**
		FC5 (5.01,0.13,0.009)	0.19 (0.03)	0.23 (0.06)	0.22 (0.05)	**0.012**	**0.042**	1.000
		FC1 (3.48,0.10,0.037)	0.25 (0.04)	0.27 (0.05)	0.29 (0.06)	0.565	**0.032**	0.504
		Cz (4.59,0.12,0.014)	0.25 (0.05)	0.28 (0.06)	0.29 (0.04)	0.087	**0.014**	1.000
		CP2 (3.84,0.10,0.026)	0.22 (0.04)	0.25 (0.06)	0.26 (0.05)	0.097	**0.032**	1.000
		P4 (4.86,0.13,0.011)	0.21 (0.03)	0.26 (0.06)	0.24 (0.05)	**0.008**	0.238	0.515
		Pz (3.95,0.11,0.024)	0.23 (0.05)	0.27 (0.05)	0.25 (0.05)	**0.020**	0.453	0.490
	Alpha	Fz (3.86,0.11,0.026)	0.26 (0.05)	0.28 (0.05)	0.25 (0.03)	0.486	0.683	**0.022**
		FC5 (4.40,0.12,0.026)	0.31 (0.05)	0.29 (0.04)	0.27 (0.04)	0.329	**0.013**	0.463
	Beta	Fz (3.51,0.10,0.036)	0.13 (0.03)	0.14 (0.03)	0.12 (0.02)	0.465	0.87	**0.031**

FoH, fear of heights; The no-FoH, m-FoH and s-FoH denote no, mild or moderate and severe fear of heights, respectively; *pcorr*, the *p* value corrected by Bonferroni. The bold values indicate that the *pcorr* values are less than 0.05.

Table 2 summarizes the statistically significant results of EEG channels in relative power between different FoH groups for microstates A, B, C and D. It was found that relative beta power of m-FoH group was higher in Fz for microstates A and C relative to s-FoH group. The results also demonstrated dramatically decreased relative alpha power for EEG signal of microstate D in multiple brain areas (F4, FC5, C3 and P7) in the s-FoH group. Furthermore, the m-FoH group had the decrease of relative delta power in frontal (F4) and frontal-central (FC6) regions for microstate C compared with the s-FoH group. Nevertheless, there were no significant differences for the comparisons of relative power of theta band between different FoH groups.

**Table 2:** Statistical results of EEG channels with significant differences in relative power of EEG rhythms between different FoH groups for microstates A, B, C and D.

Microstate type	Frequency band	Channel (F, η^2^, *p*)	Group			*pcorr*		
			no-FoH mean (std)	m-FoH mean (std)	s-FoH mean (std)	no-FoH vs. m-FoH	no-FoH vs. s-FoH	m-FoH vs. s-FoH
A	Beta	Fz (4.21,0.11,0.019)	0.15 (0.03)	0.15 (0.03)	0.13 (0.02)	1.000	0.079	**0.030**
B	Delta	FC2 (5.47,0.14,0.006)	0.24 (0.04)	0.24 (0.04)	0.28 (0.04)	1.000	**0.018**	**0.017**
		Cz (5.48,0.14,0.006)	0.24 (0.03)	0.27 (0.05)	0.28 (0.04)	0.138	**0.005**	0.533
	Alpha	FC2 (3.47,0.10,0.037)	0.29 (0.03)	0.28 (0.04)	0.26 (0.04)	1.000	**0.041**	0.207
	Beta	FC2 (4.01,0.11,0.023)	0.18 (0.03)	0.16 (0.03)	0.16 (0.02)	0.243	**0.019**	0.790
C	Delta	F4 (3.45,0.10,0.038)	0.23 (0.03)	0.21 (0.06)	0.25 (0.06)	0.393	1.000	**0.034**
		FC6 (4.26,0.11,0.018)	0.21 (0.05)	0.18 (0.04)	0.22 (0.05)	0.100	1.000	**0.024**
	Beta	Fz (6.13,0.13,0.005)	0.14 (0.03)	0.15 (0.04)	0.12 (0.02)	0.784	0.093	**0.008**
		FT10 (3.61,0.10,0.033)	0.14 (0.02)	0.16 (0.03)	0.15 (0.03)	**0.028**	0.598	0.465
D	Alpha	F4 (3.75,0.10,0.029)	0.29 (0.05)	0.30 (0.05)	0.26 (0.03)	1.000	0.210	**0.029**
		FC5 (4.57,0.14,0.016)	0.32 (0.05)	0.30 (0.04)	0.28 (0.03)	0.180	**0.016**	0.508
		C3 (3.75,0.08,0.029)	0.31 (0.03)	0.36 (0.05)	0.29 (0.03)	0.598	0.594	**0.024**
		P7 (7.76,0.15,0.011)	0.31 (0.04)	0.30 (0.05)	0.27 (0.03)	0.837	**0.003**	**0.039**

FoH, fear of heights; The no-FoH, m-FoH and s-FoH denote no, mild or moderate and severe fear of heights, respectively; *pcorr*, the *p* value corrected by Bonferroni. The bold values indicate that the *pcorr* values are less than 0.05.

### 3.2 Comparisons of power ratios of microstate EEG rhythms between different FoH groups

The distributions of brain topographic maps for average power ratios of basic EEG rhythms in different FoH groups for EEG signals of microstate D are displayed in Figure 4 (see Supplementary Figures 5–8 for brain topographies of power ratios for other micsrostate EEG signals). From the figure, the scalp distributions of each type of power ratio were broadly similar, showing higher values in the frontal and central regions than in other brain regions. Particularly, s-FoH group exhibited heightened power ratio of theta to beta when contrasted with both no-FoH and m-FoH groups.

**Figure 4:**
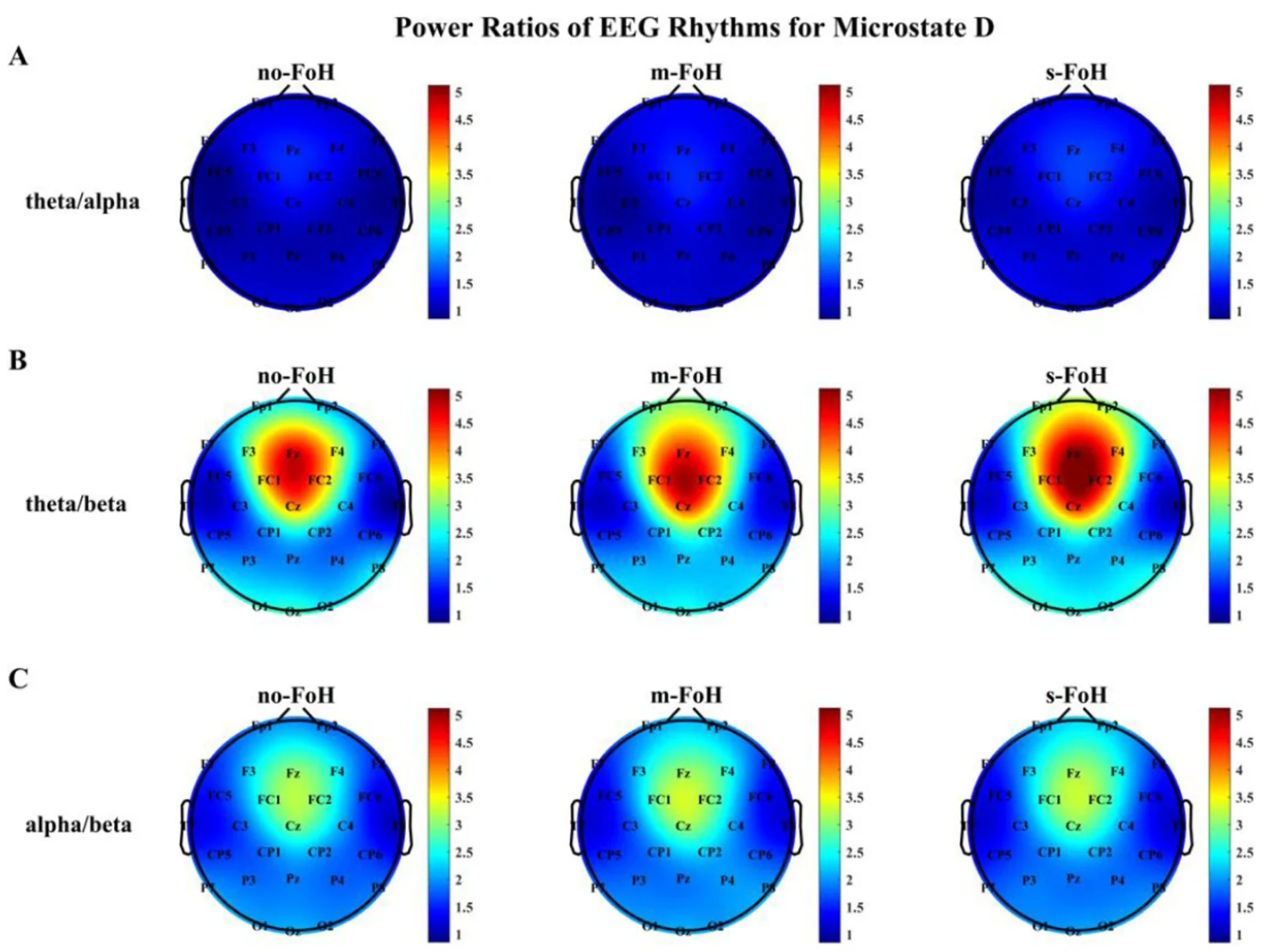
Distributions of brain topographic maps for average power ratios of EEG rhythms of different FoH groups for microstate D: **(A)** theta/alpha, **(B)** theta/beta, and **(C)** alpha/beta. Each row of each subgraph represents the average power ratio results of the no-FoH, m-FoH and s-FoH groups based on EEG signals of microstate D. For the explanation of the abbreviations in the figure, please refer to Figure 3.

The statistical results of EEG channels with significant differences in power ratios of EEG rhythms for five classes of microstates among the three groups are presented in Table 3. We observed prominently enhanced power ratio of theta to alpha in frontal-central areas for multiple microstates (microstate B: FC2; microstates C, D and F: FC5) and theta to beta in Fp2 for microstate D in the s-FoH group as compared to the no-FoH group. Meanwhile, remarkably higher power ratio theta/alpha of m-FoH group was found in the central region (Cz) for microstate A and the parietal area (P7) for microstate D in relation to no-FoH group. In addition, s-FoH group had a significant increase in theta/alpha (microstate D: C3) and theta/beta (microstate C: Fz; microstate F: F4) than m-FoH group. From these results, we can see that no consistent monotonic increase in these power ratios was observed across all microstates, channels, and group comparisons.

**Table 3:** Statistical results of EEG channels with significant differences in power ratios of EEG rhythms between different FoH groups for microstates A, B, C, D and F.

Microstate type	Power ratio	Channel (*F*, η^2^, *p*)	Group			*pcorr*		
			no-FoH mean (std)	m-FoH mean (std)	s-FoH mean (std)	no-FoH vs. m-FoH	no-FoH vs. s-FoH	m-FoH vs. s-FoH
A	Theta/alpha	Cz (3.89,0.11,0.025)	1.06 (0.27)	1.29 (0.29)	1.23 (0.29)	**0.026**	0.138	1.000
B	Theta/alpha	FC2 (3.26,0.10,0.045)	1.01 (0.22)	1.12 (0.23)	1.19 (0.24)	0.338	**0.040**	0.957
C	Theta/alpha	FC5 (3.74,0.06,0.032)	0.89 (0.20)	1.00 (0.36)	1.05 (0.17)	0.520	**0.027**	0.883
	Theta/beta	Fz (3.21,0.09,0.047)	2.46 (0.66)	2.29 (0.77)	2.80 (0.67)	1.000	0.362	**0.045**
D	Theta/alpha	FC5 (3.81,0.10,0.027)	0.85 (0.22)	0.98 (0.25)	1.03 (0.18)	0.186	**0.025**	1.000
		C3 (4.13,0.11,0.020)	0.99 (0.16)	0.93 (0.27)	1.12 (0.23)	1.000	0.248	**0.018**
		CP5 (3.52,0.10,0.035)	0.94 (0.21)	1.00 (0.27)	1.12 (0.21)	1.000	**0.036**	0.248
		P7 (4.01,0.11,0.023)	1.08 (0.29)	1.17 (0.30)	1.32 (0.26)	**0.022**	0.924	0.205
	Theta/beta	Fp2 (3.46,0.10,0.037)	1.99 (0.70)	2.68 (1.40)	3.00 (1.51)	0.236	**0.035**	1.000
F	Theta/alpha	FC5 (3.40,0.09,0.039)	0.91 (0.21)	0.96 (0.24)	1.10 (0.32)	1.000	**0.049**	0.170
	Theta/beta	F4 (4.13,0.11,0.020)	1.78 (0.47)	1.49 (0.45)	1.96 (0.74)	0.286	0.953	**0.018**

FoH: fear of heights; The no-FoH, m-FoH and s-FoH denote no, mild or moderate and severe fear of heights, respectively; *pcorr*: the *p* value corrected by Bonferroni. The bold values indicate that the *pcorr* values are less than 0.05.

### 3.3 Correlations between microstate EEG power features and AQ scores

The correlations between AQ-anxiety scores and relative power of EEG rhythms in different brain regions for microstate F are illustrated in Figure 5. The results showed that AQ-anxiety scores were significantly positively related to relative delta power without *p* values corrected by Bonferroni but had no correlations with the correction of *p* values (i.e. *pcorr*) in frontal (Fz: *r* = 0.273, *p*/*pcorr* = 0.025/0.200), frontal-central (FC1: *r* = 0.291, *p*/*pcorr* = 0.017/0.136), central (Cz: *r* = 0.284, *p*/*pcorr* = 0.020/0.16) and central-parietal (CP2: *r* = 0.292, *p*/*pcorr* = 0.017/0.136) regions, but had a negative relationship with relative alpha power in frontal-central (FC5: *r* = −0.319, *p* = 0.009) area. However, no significant differences were observed between AQ-avoidance scores and relative power of microstate EEG.

**Figure 5:**
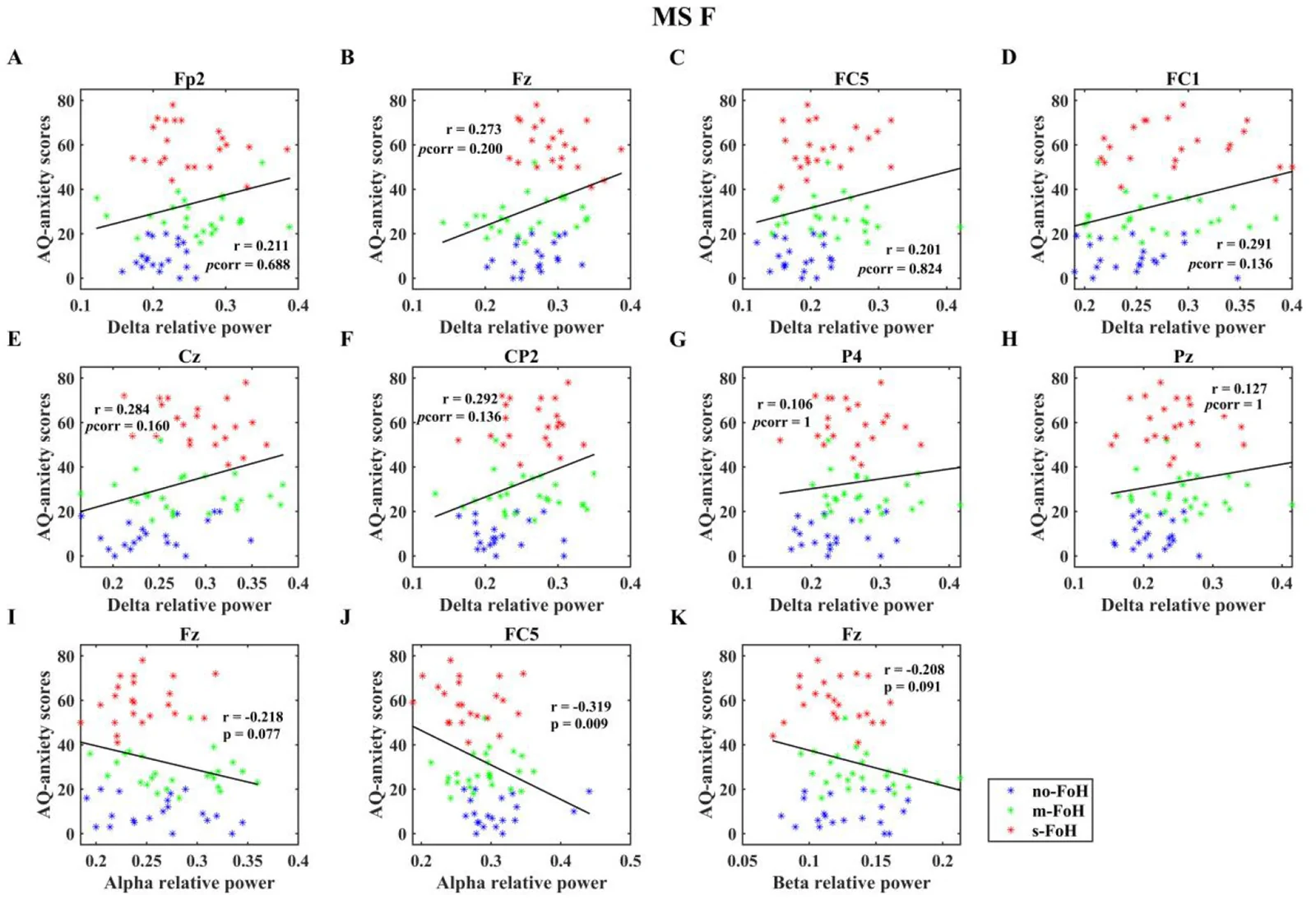
Correlations between AQ-anxiety scores and relative power of EEG rhythms in different brain regions for microstate F. **(A)** Fp2, **(B)** Fz, **(C)** FC5, **(D)** FC1, **(E)** Cz, **(F)** CP2, **(G)** P4, **(H)** Pz, **(I)** Fz, **(J)** FC5, and **(K)** Fz. *pcorr*: the *p* value corrected by Bonferroni (for the correlation analysis of the same EEG relative power feature involving more than three brain regions). For the explanation of the abbreviations in the figure, please refer to Figure 3.

Table 4 describes the correlation results of AQ scores and relative power for EEG signals of microstates A–D. It can be observed from the table that AQ-anxiety (AQ-avoidance) scores negatively correlated with relative power of alpha and beta bands for all microstates but showed a significantly positive association with relative delta power for microstates B and C, which mainly occurred in the frontal and frontal-central regions.

**Table 4:** Correlation results between acrophobia questionnaire (AQ) scores and relative power of EEG rhythms in brain regions for microstates A, B, C and D.

Microstate type	Channel (band)	AQ-anxiety		AQ-avoidance	
		*r*	*p*/*pcorr*	*r*	*p*/*pcorr*
A	Fz (beta)	−0.280	0.022/-	−0.289	0.017/-
B	FC2 (delta)	0.257	0.036/-	0.356	0.003/-
	Cz (delta)	0.292	0.016/-	0.441	0.000/-
	FC2 (alpha)	−0.250	0.041/-	−0.260	0.033/-
	FC2 (beta)	−0.324	0.008/-	−0.312	0.010/-
C	F4 (delta)	0.225	0.067/-	0.257	0.036/-
	FC6 (delta)	0.128	0.302/-	0.188	0.128/-
	Fz (alpha)	−0.208	0.091/-	−0.245	0.046/-
	FT10 (beta)	0.080	0.518/-	−0.059	0.638/-
D	F4 (alpha)	−0.235	0.055/0.220	−0.247	0.044/0.176
	FC5 (alpha)	−0.312	0.010/0.040	−0.305	0.012/0.048
	C3 (alpha)	−0.270	0.027/0.108	−0.146	0.237/0.948
	P7 (alpha)	−0.367	0.002/0.008	−0.423	0.000/0.000

For analyses involving more than three brain regions, raw and Bonferroni-corrected *p*-values were reported as *p*/*pcorr*. Only raw *p*-values were shown for analyses with one or two brain regions, where no multiple comparison correction was performed.

The correlation results between AQ scores and power ratios of EEG rhythms of different microstates are summarized in Table 5. Significantly positive correlations in the table were found between the score of AQ-anxiety or avoidance and the power ratio of theta to alpha for microstate D in parietal area. Moreover, the power ratio theta/alpha in frontal-central area (microstate B: FC2; microstate F: FC5) and central area (microstate A: Cz) also positively correlated with AQ scores. As regards microstate C, there existed significant negative associations between the power ratio of theta to beta and the AQ-anxiety and avoidance scores. In combination with the above results, it was manifested that microstate EEG power characteristics, especially relative delta and alpha power and the power ratio of theta to alpha extracted from frontal and frontal-central electrical activity, were closely associated with the degree of fear of heights, which has potential application value for the auxiliary diagnosis of fear of heights.

**Table 5:** Correlation results between acrophobia questionnaire (AQ) scores and power ratios of EEG rhythms in brain regions for microstates A, B, C, D and F.

Microstate type	Channel (power ratio)	AQ-anxiety		AQ-avoidance	
		*r*	*p*/*pcorr*	*r*	*p*/*pcorr*
A	Cz (theta/alpha)	0.211	0.086/-	0.280	0.022/-
B	FC2 (theta/alpha)	0.272	0.026/-	0.246	0.045/-
C	FC5 (theta/alpha)	0.196	0.112/-	0.175	0.156/-
	Fz (theta/beta)	−0.248	0.043/-	−0.269	0.028/-
D	F4 (theta/alpha)	0.250	0.041/0.205	0.241	0.049/0.245
	FC5 (theta/alpha)	0.253	0.039/0.195	0.249	0.042/0.210
	C3 (theta/alpha)	0.302	0.013/0.065	0.186	0.132/0.660
	CP5 (theta/alpha)	0.259	0.034/0.170	0.238	0.053/0.265
	P7 (theta/alpha)	0.344	0.004/0.020	0.368	0.002/0.010
	Fp2 (theta/beta)	0.299	0.014/-	0.230	0.061/-
F	FC5 (theta/alpha)	0.290	0.017/-	0.208	0.090/-
	F4 (theta/beta)	0.173	0.161/-	0.066	0.594/-

For analyses involving more than three brain regions, raw and Bonferroni-corrected *p*-values were reported as *p*/*pcorr*. Only raw *p*-values were shown for analyses with one or two brain regions, where no multiple comparison correction was performed.

### 3.4 Comparisons of microstate functional connectivity strength between different FoH groups

The number of microstate windows and the cumulative signal duration used for wPLI estimation for each subject are provided in Supplementary Tables 1, 2, respectively. The Kruskal–Wallis one-way ANOVA analysis showed that three groups had significant differences in the number of microstate windows (H = 12.32, *p* = 0.002) and the cumulative signal duration (H = 12.77, *p* = 0.002) for microstate F. The multiple comparison results further displayed that compared to the m-FoH and s-FoH groups, the no-FoH group had a greater number of microstate windows (no-FoH vs. m-FoH: H = 20.00, *pcorr* = 0.003; no-FoH vs. s-FoH: H = 16.83, *pcorr* = 0.017) and a longer cumulative signal time (no-FoH vs. m-FoH: H = 20.33, *pcorr* = 0.002; no-FoH vs. s-FoH: H = 17.20, *pcorr* = 0.014). Figure 6 depicts the average functional connectivity matrices of different FoH groups for five types of microstates. It could be observed that these association matrices followed a similar pattern in all three groups for the same kind of microstate but showed different patterns for different classes of microstates. Intuitively, the mean connection strength of microstate F was smaller than that of other microstates. Then, we computed and compared the average FCS of whole brain area, intra-region and inter-region for four seven sub-regions between the groups with different FoH.

**Figure 6:**
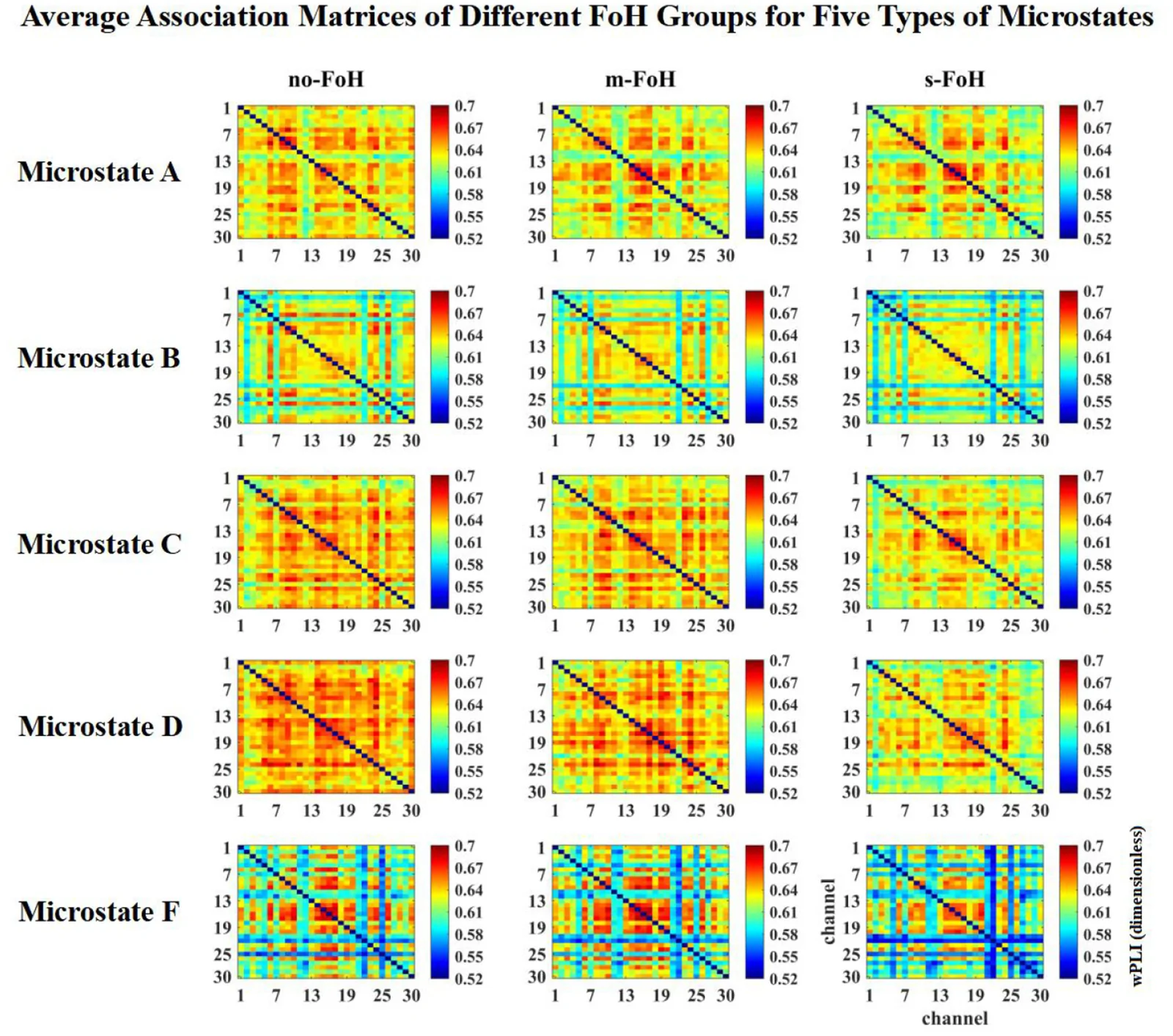
Average association matrices of different FoH groups for microstates A, B, C, D and F. Each row of each subgraph represents the average functional connectivity strength of the no-FoH, m-FoH and s-FoH groups. FoH: fear of heights; the no-FoH, m-FoH and s-FoH denote no, mild or moderate and severe fear of heights, respectively; wPLI: weighted phase lagged index. wPLI is a dimensionless metric with a theoretical range of 0 to 1. For visualization purposes, the color bar is truncated to the value range of 0.52 to 0.7 observed in the present study. The color bar indicates wPLI value (dimensionless; plotted range: 0.52–0.7, theoretical range: 0–1). The labels 1–30 denote the EEG channels Fp1, Fz, F3, F7, FT9, FC5, FC1, C3, T7, CP5, CP1, Pz, P3, P7, O1, Oz, O2, P4, P8, CP6, CP2, Cz, C4, T8, FT10, FC6, FC2, F4, F8 and Fp2, respectively.

The results of ANOVA indicated that there existed significant differences in the connection strength of the right-upper and left-lower brain regions (*F*_2, 66_ = 3.545, η^2^ = 0.10, *p* = 0.034), the right-upper and midline brain regions (*F*_2, 66_ = 3.435, η^2^ = 0.09, *p* = 0.038) as well as within the midline brain regions (*F*_2, 66_ = 3.642, η^2^ = 0.10, *p* = 0.032) for microstate A among the three groups. Multiple comparison tests further demonstrated that the no-FoH group showed significantly stronger FCS than the s-FoH group (the FCS of the no-FoH (s-FoH) groups were 0.654 ± 0.036 (0.620 ± 0.029), 0.634 ± 0.031 (0.611 ± 0.027) and 0.634 ± 0.030 (0.605 ± 0.037), respectively; the corrected *p* values were 0.038, 0.033 and 0.031, respectively). The ANOVA analysis also revealed significant group differences in FCS of intra-region for the right-upper brain regions (*F*_2, 66_ = 4.064, η^2^ = 0.11, *p* = 0.022), the right-upper and left-lower brain areas (*F*_2, 66_ = 3.923, η^2^ = 0.11, *p* = 0.025), and the right-upper and right-lower brain areas (*F*_2, 66_ = 4.345, η^2^ = 0.06, *p* = 0.017) for microstate F. The Bonferroni *post*-*hoc* test was performed and the results showed that the m-FoH group had prominently larger FCS for all three cases in comparison with individuals with severe fear of heights (the FCS of the m-FoH (s-FoH) groups were 0.601 ± 0.038 (0.527 ± 0.031), 0.621 ± 0.031 (0.598 ± 0.023) and 0.620 ± 0.032 (0.595 ± 0.025), respectively; the corrected *p* values were 0.029, 0.025 and 0.015, respectively).

### 3.5 Comparisons of basic properties of microstate functional brain networks between different FoH groups

Figure 7 illustrates the comparative results of basic network metrics between different FoH groups with network sparsity ranging from 10% to 50% for microstate F. For functional brain network of microstate F, the no-FoH group exhibited notably decreased *C* and *E*_*loc*_ compared to the m-FoH and s-FoH groups under a wide sparsity range. The results also showed that the values of γ and normalized *E*_*loc*_ in the m-FoH group were higher than the no-FoH and s-FoH groups for a few sparsity values. With respect to functional brain network of microstate A (see Supplementary Figure 9), we observed that relative to the no-FoH group, the s-FoH group had a significant increase of λ but decrease of normalized *E*_*glo*_for a number of sparsity values; on the contrary, the s-FoH group showed significantly decreased λ and increased normalized *E*_*glo*_than the m-FoH group under several other sparsity values. As regards functional brain network of microstate C (see Supplementary Figure 10), it was found that there were significant differences in *C* and *E*_*loc*_ between the no-FoH and m-FoH groups with a sparsity of 10%−30%, where the latter was significantly larger than the former. Besides, as for the functional brain network of microstate D (see Supplementary Figure 11), the results displayed a prominently reduced *C* and *E*_*loc*_ in the s-FoH group under multiple sparsity values in the range of 10% to 30%. However, we did not find any significant differences in the topological properties of functional brain network for microstate B among the three groups. In short, the differences in network attributes between no-FoH and FoH groups mainly occurred in the functional brain network of microstate F. Furthermore, the small-world characteristics γ and λ of microstate functional brain networks in the three groups had values closer to 1 with comparable traits of the random network as well as the normalized *E*_*glo*_ and normalized *E*_*loc*_, suggesting that an optimal balance between local specialization and global integration of normal brain network was disrupted during the virtual high-altitude exposure and the brain networks tended to be a shift toward randomization.

**Figure 7:**
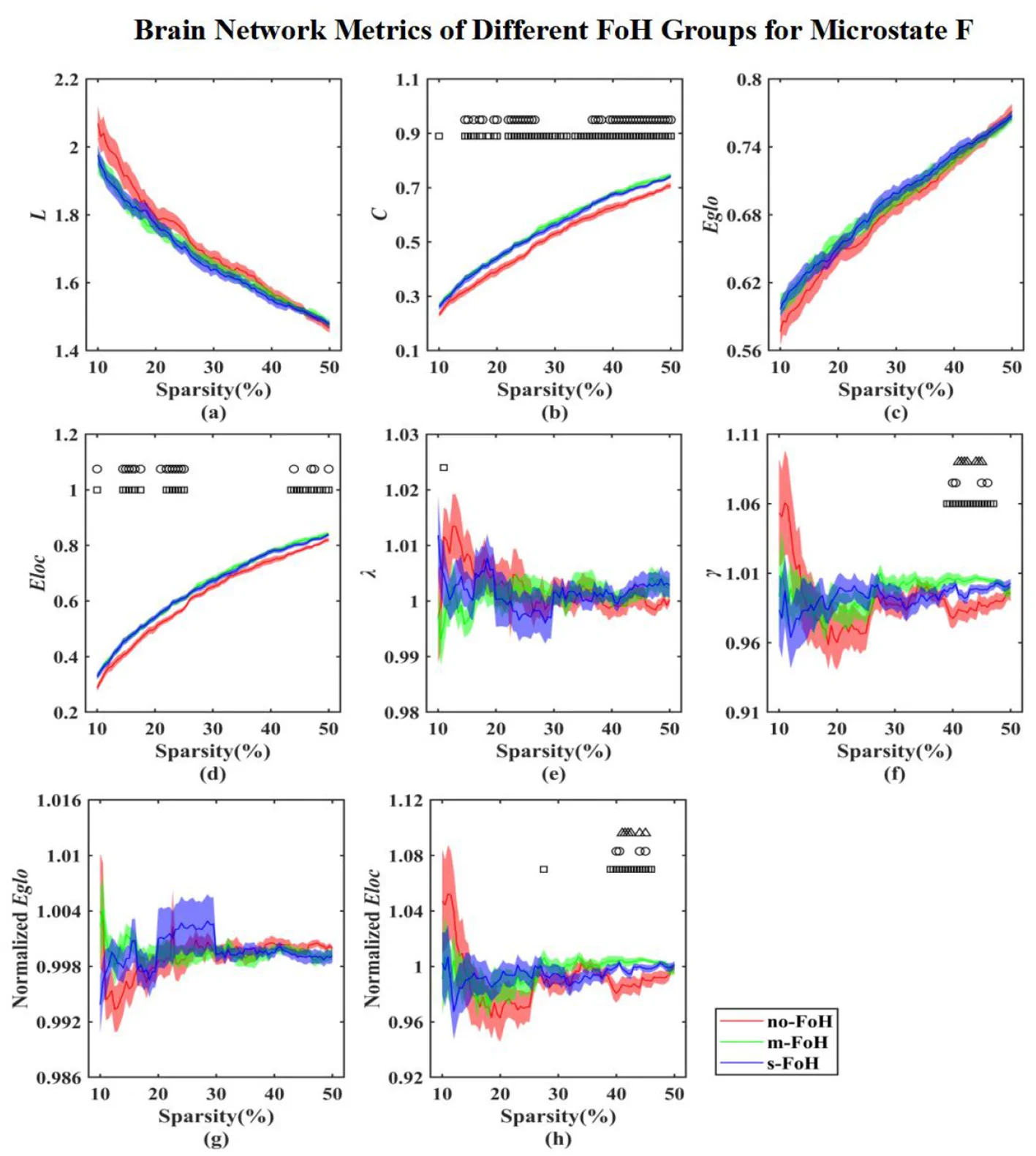
Comparison results of brain network properties of microstate F as a function of sparsity for different FoH groups. The network metrics (a) *L*, (b) *C*, (c) *E*_*glo*_, (d) *E*_*loc*_, (e) λ, (f) γ, (g) normalized *E*_*glo*_ and (h) normalized *E*_*loc*_ denote characteristic path length, clustering coefficient, global efficiency, local efficiency, normalized characteristic path length, normalized clustering coefficient, normalized global efficiency and normalized local efficiency, respectively. Shaded areas around the mean values represent the standard errors. The squares □ indicate significant differences between the no-FoH and m-FoH groups, the circles ° for significant differences of the no-FoH and s-FoH groups, and the triangles Δ for significant differences of the m-FoH and s-FoH groups (*p* < 0.05, Bonferroni correction). FoH: fear of heights; the no-FoH, m-FoH and s-FoH denote no, mild or moderate and severe fear of heights, respectively.

It was clear that the significant differences of the topological properties of the microstate brain networks between individuals with different degrees of FoH were mainly closely related to the microstate F. So, for each network metric of the microstate brain network F, the Functional Data analysis was employed for analyzing the differences of group mean graph curves. The statistical results are shown in Table 6. Consistent with the results of Figure 7, the areas of mean graph curves between no-FoH and FoH groups for network metrics *C* and *E*_*loc*_both were significantly larger than expected in the null distribution, and the graph curves of m-FoH and s-FoH groups showed no significant differences of all network metrics.

**Table 6:** The statistical results for the area between group mean graph curves for microstate brain network F under the sparsity range of 10%−50% based on the Functional Data analysis.

Network metrics	Area between group mean graph curves		
	no-FoH and m-FoH (Area, *pcorr*)	no-FoH and s-FoH (Area, *pcorr*)	m-FoH and s-FoH (Area, *pcorr*)
*L*	(2.707, 0.183)	(2.990, 0.134)	(0.855, 0.951)
*C*	(3.684, **<** **0.001**)	(3.109, **0.006**)	(0.615, 0.813)
*E_*glo*_*	(0.685, 0.425)	(0.774, 0.303)	(0.283, 0.949)
*E_*loc*_*	(2.636, **0.002**)	(2.347, **0.005**)	(0.417, 0.987)
λ	(0.268, 0.343)	(0.260, 0.597)	(0.214, 0.688)
γ	(1.350, 0.114)	(1.408, 0.118)	(0.778, 0.517)
Normalized *E_*glo*_*	(0.113, 0.367)	(0.143, 0.478)	(0.114, 0.654)
Normalized *E_*loc*_*	(1.131, 0.139)	(1.186, 0.124)	(0.595, 0.716)

FoH: fear of heights; *L, C, E*_*glo*_, *E*_*loc*_, λ, γ, normalized *E*_*glo*_ and normalized *E*_*loc*_ denote characteristic path length, clustering coefficient, global efficiency, local efficiency, normalized characteristic path length, normalized clustering coefficient, normalized global efficiency and normalized local efficiency, respectively. *pcorr*: the *p* value corrected by Bonferroni. The bold values indicate that the *pcorr* values are less than 0.05.

### 3.6 Degree distributions of microstate functional brain networks for different FoH groups

Figures 8A, B respectively exhibit the histograms of regional degree distributions for different FoH groups and the curves of the logarithm of degree vs. the logarithm of cumulative probability of degree under three degree distribution forms for functional brain network of microstate A. As illustrated in the figure, the best-fitting model was the exponentially truncated power law for the degree distribution of the no-FoH group (i.e., *P*(*k*) ~ *k*^α−1^e^k/*kc*^, where α = −1.701, *kc* = 0.183) with coefficient of determination *R*^2^ = 0.9772, while the degree distributions for both the m-FoH and s-FoH groups conformed to the power law pattern (i.e., *P*(*k*) ~ *k*^α^−1, where α = 5.923, 8.261, respectively) with the values of *R*^2^ = 0.9800, 0.9941, respectively. However, the degree distributions of functional brain networks of microstate D in individuals with different levels of FoH were just inverse (Figure 9); that is to say, the degree distribution of the no-FoH group showed the power law form (α = 6.377, *R*^2^ = 0.9527) and the degree distributions of m-FoH and s-FoH groups had the form of exponentially truncated power law (α = 5.555, −2.472; *kc* = 0.686, 0.190; *R*^2^ = 0.9959, 0.9930; respectively). In addition, the results also indicated that in terms of all the groups, the degree distributions in brain network of microstate B was best fitted by the power law (see Supplementary Figure 12), whereas the degree distributions of brain networks of microstates C and F followed exponentially truncated power law (see Supplementary Figures 13, 14).

**Figure 8:**
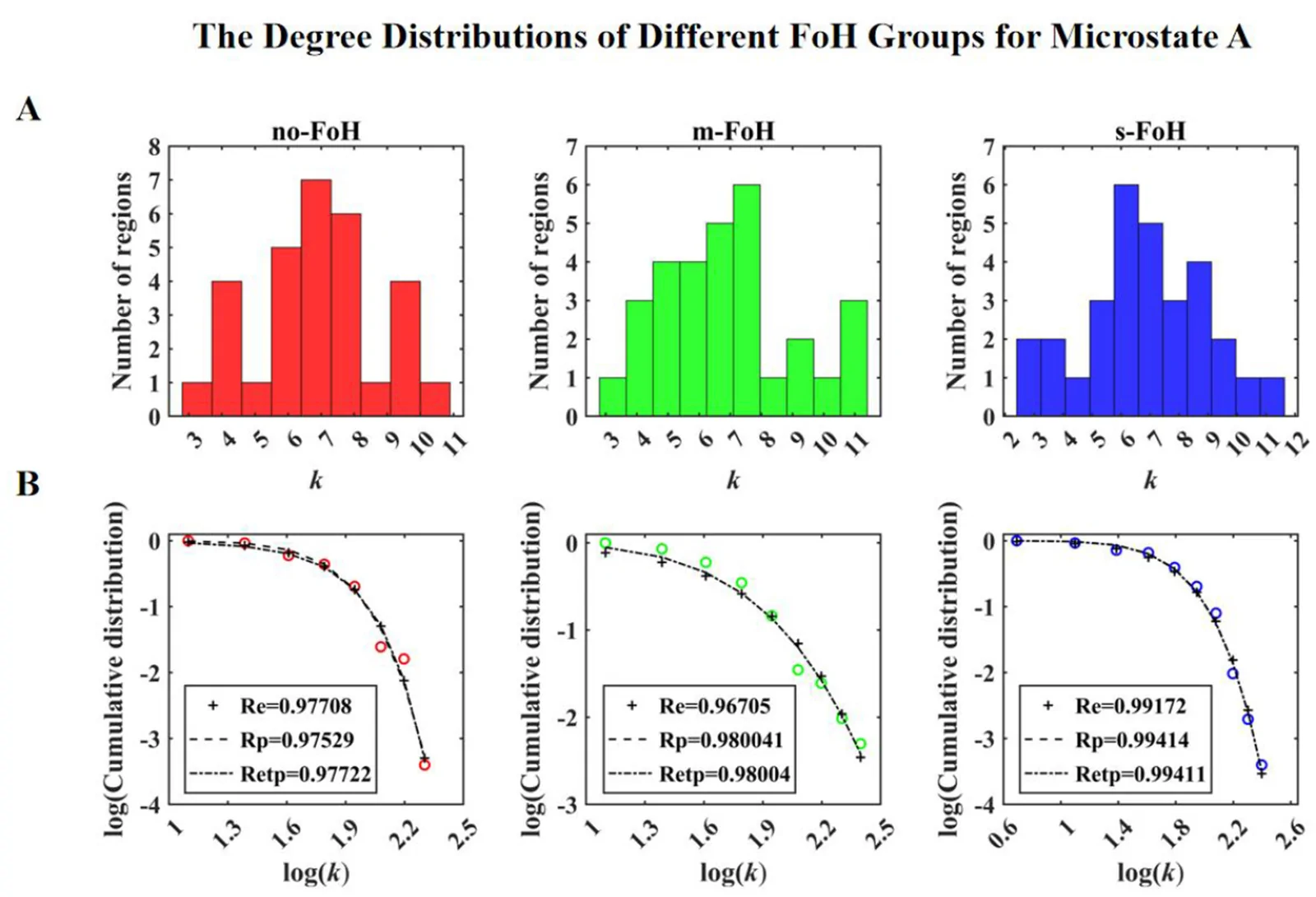
The degree distributions of functional brain networks of microstate A for different FoH groups. **(A)** The histograms of regional degree *k* distributions for different FoH groups, and **(B)** the fitted curves of the logarithm of degree log(*k*) vs. the logarithm of cumulative probability of degree under three degree distribution forms. The red circle 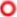 represents the observation data of the no-FoH group, the green circle 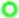 for the observation data of the m-FoH group, and the blue circle 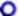 for the observation data of the s-FoH group. The sign **+** indicates the fit of an exponential (*P*(*k*) ~ *e*^*ak*^), the dashed line -- for the fit of a power law (*P*(*k*) ~ *k*^α^−1), and the dotted line -. for the fit of an exponentially truncated power law (*P*(*k*) ~ *k*^α^−1e^k/*kc*^). Re denotes the coefficient of determination *R*^2^ for the exponential fit, Rp for the power law fit, and Retp for the exponentially truncated power-law fit. FoH, fear of heights; the no-FoH, m-FoH and s-FoH denote no, mild or moderate and severe fear of heights, respectively. According to the estimated *R*^2^ values, the degree distribution of the no-FoH group was best fitted by the exponentially truncated power law (α = −1.701, *kc* = 0.183), and the power law distribution was more suitable for the degree distributions of the m-FoH and s-FoH groups (m-FoH: α = 5.923; s-FoH: α = 8.261).

**Figure 9:**
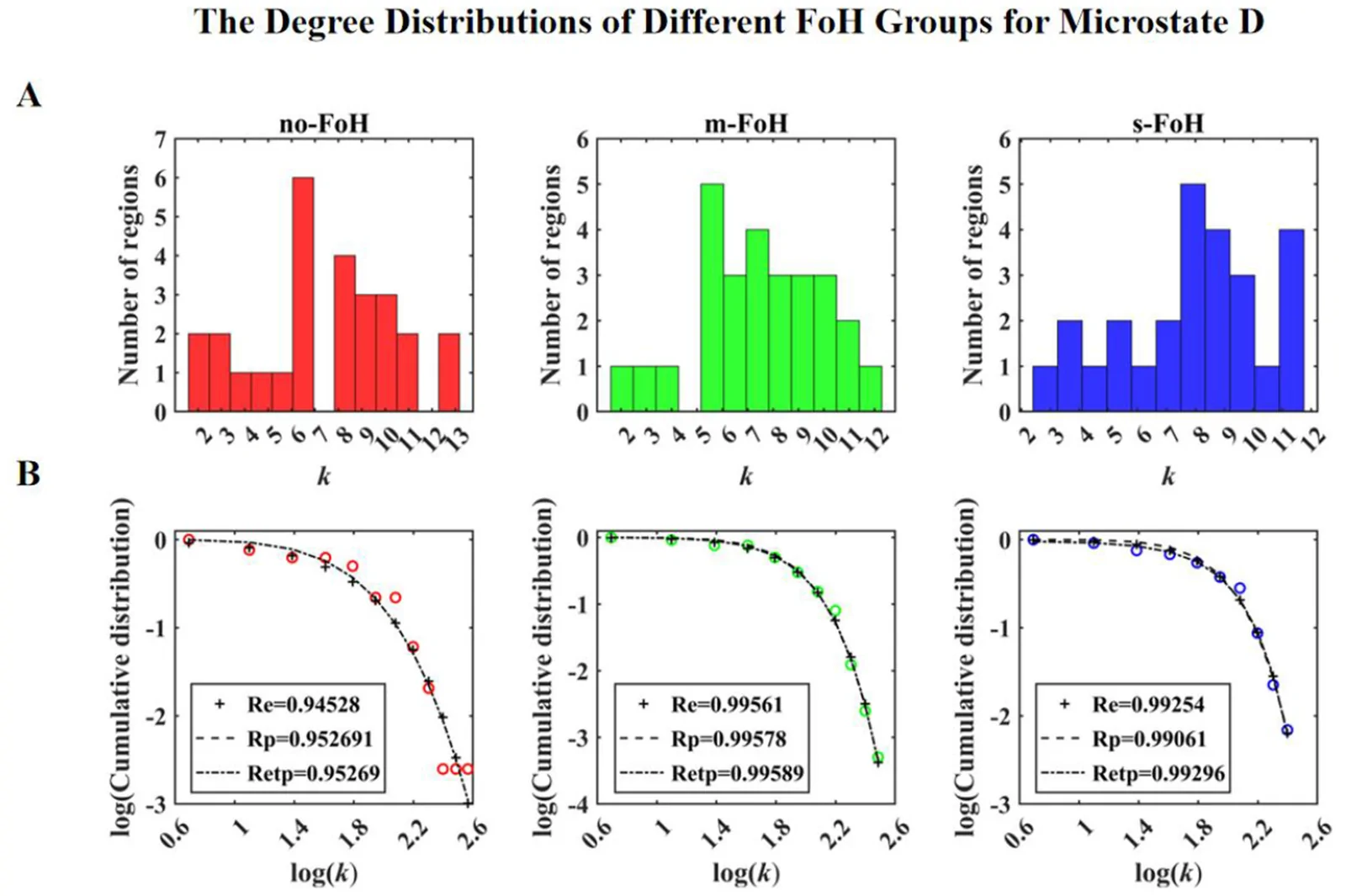
The degree distributions of functional brain networks of microstate D for different FoH groups. **(A)** The histograms of regional degree *k* distributions for different FoH groups, and **(B)** the fitted curves of the logarithm of degree log(*k*) vs. the logarithm of cumulative probability of degree under three degree distribution forms. For the explanation of the abbreviations in the figure, please refer to Figure 8. According to the estimated *R*^2^ values, the degree distribution of the no-FoH group was best fitted by the power law (α = 6.377), and the exponentially truncated power law distribution was more suitable for the degree distributions of the m-FoH and s-FoH groups (m-FoH: α = 5.555, *kc* = 0.686; s-FoH: α = 8.261, *kc* = 0.190).

### 3.7 Hub nodes of microstate functional brain networks for different FoH groups

Figure 10 displays hub nodes in the functional brain networks of different FoH groups for five types of microstates. As shown in Figure 10A, the temporal (T7 and T8 for three FoH groups) and the occipital (no-FoH: O1; m-FoH: O1 and O2; s-FoH: O2) regions were identified as common hub nodes in the functional brain network of microstate A. Besides, the frontal area Fp2 was only identified as a hub for the no-FoH group. As for microstate B (Figure 10B), the commonly recognized hubs included temporal (T7), parietal (P7) and occipital (O2) regions for three groups, central-parietal (CP5) and central (C4) areas for no-FoH and m-FoH groups, and temporal region T8 for no-FoH and s-FoH groups. Likewise, the frontal Fp2 was only the hub node of the no-FoH group. Furthermore, the s-FoH group had the occipital O1 and Oz acting as hubs. With regards to microstate C (Figure 10C), the distribution of hub nodes in the no-FoH group was mainly concentrated in the left cerebral hemisphere with left-lateralization, but the hub distributions for both m-FoH and s-FoH groups were relatively uniform in the left and right brain hemispheres. Some of all identified hubs in the three groups were distributed in the temporal (T7 and T8), central-parietal (CP5 and CP6) and occipital (O1, Oz and O2) areas. In addition, the frontal-central FC5 and frontal Fp2 in the no-FoH group were identified as hub nodes and the parietal P7 and P8 were recognized as hubs in the s-FoH group. For microstate D (Figure 10D), the number of hub nodes in the three groups was relatively small as compared with other microstate brain networks, but the hub distributions of different FoH groups were basically consistent, mainly including the central-frontal (FC5 and FC6) and central (C3) regions. Moreover, the no-FoH and m-FoH groups had the network hub in the central-parietal (CP5) region, while the temporal (T8) region acted as the key node for the s-FoH group. Finally, as regards to microstate F (Figure 10E), it was found that the same hub nodes in different FoH groups were located in the occipital (O1 and O2) and central-parietal (CP5) areas. The results also showed that the parietal region was identified as the network hub for the FoH groups (m-FoH: P7, s-FoH: P8).

**Figure 10:**
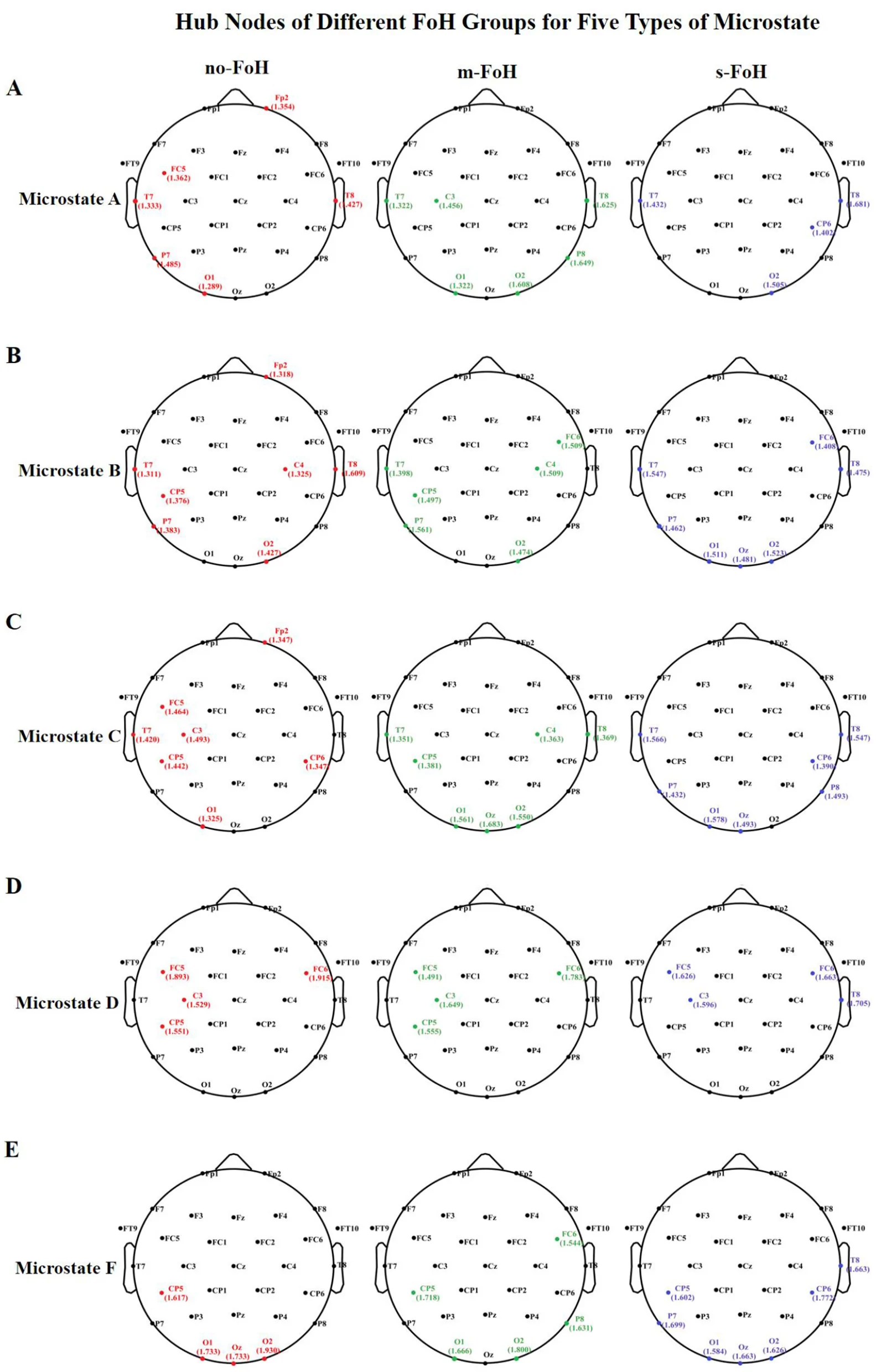
The hub nodes in functional brain networks and their normalized degree values for the no-FoH, m-FoH and s-FoH groups for five types of microstates. **(A)** microstate A, **(B)** microstate B, **(C)** microstate C, **(D)** microstate D, and **(E)** microstate F. The red nodes 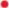 were identified as hubs of brain network in the no-FoH group, the green nodes 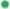 were identified as hubs of brain network in the m-FoH group, and the blue nodes 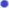 were identified as hubs of brain network in the s-FoH group. FoH: fear of heights; the no-FoH, m-FoH and s-FoH denote no, mild or moderate and severe fear of heights, respectively.

### 3.8 Comparisons of robustness of microstate functional brain networks between different FoH groups

Figure 11 illustrates the robustness of functional brain networks of microstate F among different FoH groups in response to the targeted attack or random failure. As can be seen from the figure, the significant differences were observed between the no-FoH and m-FoH groups and between the no-FoH and s-FoH groups. Specifically, under the targeted attack condition (Figures 11A, C), the no-FoH group exhibited significantly larger relative size of the largest cluster in brain network than the FoH groups during the selective elimination of 10% to 60% of nodes; global efficiency in the no-FoH group was also dramatically greater than that in the m-FoH group for nodes removal from 20% to 50%. On the other hand, when nodes were randomly eliminated from 50% to 90%, prominently larger relative size of largest cluster in the no-FoH group was found for the random failure condition in comparison with the FoH groups (Figures 11B, D). Additionally, there were almost no significant differences in brain network robustness between individuals with different degrees of FoH for other microstates. Therefore, the no-FoH group showed stronger the robustness of microstate functional brain network FoH groups, which was closely associated with microstate F.

**Figure 11:**
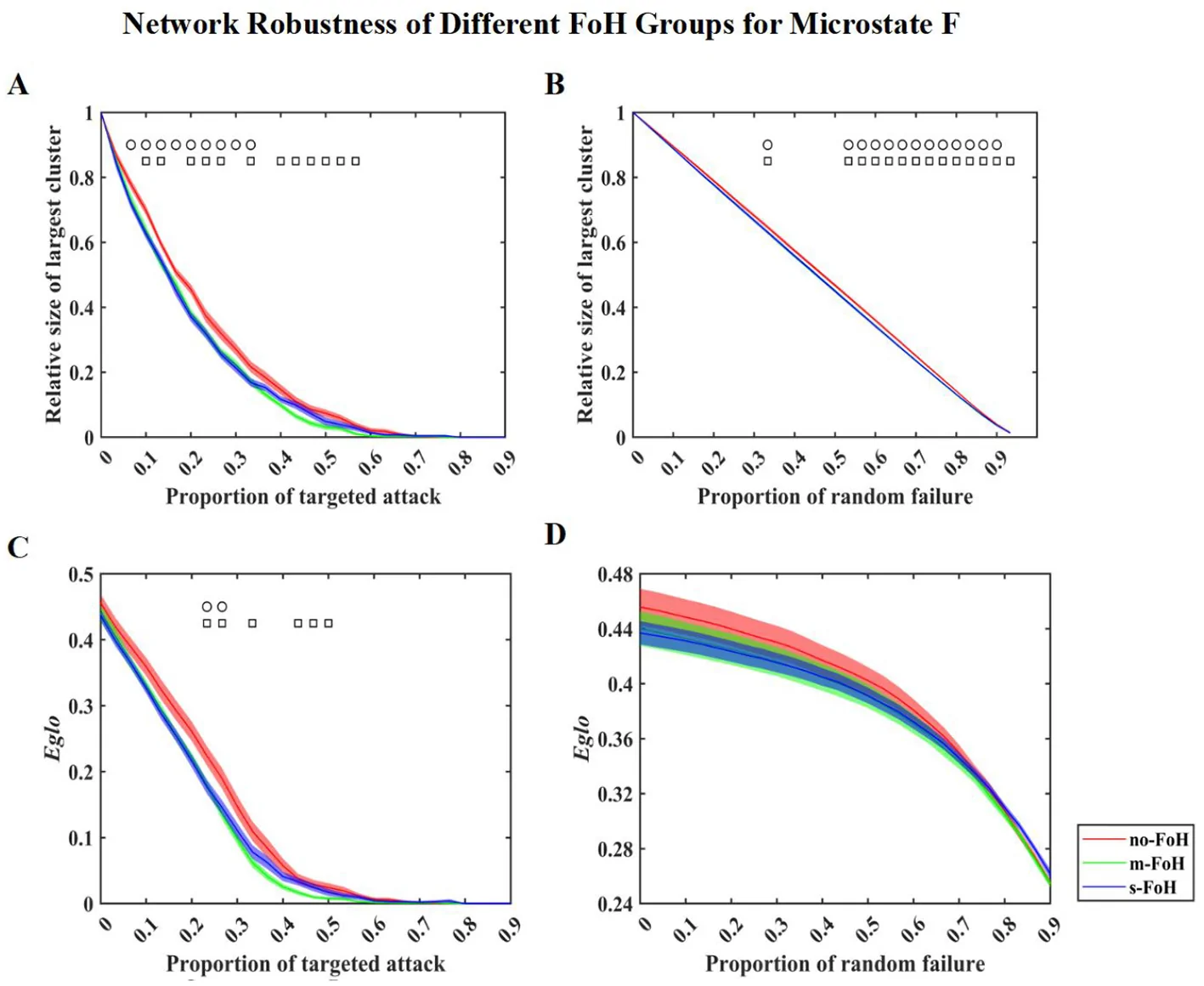
The brain network robustness of different FoH groups for microstate F in response to the targeted attack and random failure. **(A, C)** denote relative size of largest cluster and global efficiency of functional brain networks as a function of proportion of removed nodes by targeted attack, respectively. **(B, D)** represent relative size of largest cluster and global efficiency of functional brain networks as a function of proportion of removed nodes by random failure, respectively. For the explanation of the abbreviations in the figure, please refer to Figure 7.

## 4 Discussion

In this study, we combined EEG microstate, Welch, and wPLI approaches to investigate brain function activity in people with different severity of FoH during virtual high-altitude exposure scene. We found that the s-FoH group showed significantly higher relative power of delta for microstate F and power ratio theta/alpha for microstates B, C, D and F, where the increases were most prominent in the frontal, frontal-central and central regions. Our results also indicated that the no-FoH and m-FoH groups had a significant increase in relative alpha power for microstates D, F compared to the s-FoH group. Besides, AQ scores showed notably positive relationships with relative delta power and power ratio theta/alpha but negative correlations with relative alpha power for multiple microstates in multiple brain areas. Moreover, the functional brain networks for microstate F in both m-FoH and s-FoH groups manifested dramatically increased clustering coefficient and local efficiency but reduced robustness. Specifically, the degree distributions of acrophobic FoH and no-FoH groups exhibited different patterns in microstates A and D, respectively. In addition, the hub nodes of brain networks in different FoH groups also varied in each type of microstate.

### 4.1 Altered power spectra of microstate EEG in people with severe fear of heights under the exposure of virtual high-altitude

EEG power spectrum analysis has conventionally been used to probe the power of different brain areas, reflecting the intensity of brain function activity (Cheung et al., 2018; Imperatori et al., 2015; Cao et al., 2016; Case et al., 2018; Das et al., 2024). In general, the dominant rhythmic EEG oscillations vary under different brain states. For example, the slowest delta waves observed in different states of awareness (e.g., anesthesia, fatigue and meditation) were most prominent during deep sleep (Hossain et al., 2024). Further, EEG oscillatory activities were also affected by psychiatric illnesses and significantly altered as compared to healthy controls. For example, in a recent EEG study, it was reported that patients with borderline personality disorder exhibited a greater reduction of alpha power in posterior brain region (Deiber et al., 2024). Since different types of microstates were considered as representing different psychological or mental states (Lehmann and Koenig, 1997), they respectively reflected the specific functional configuration patterns of the brain within approximately several tens of milliseconds. It is intuitively clear that different types of microstate EEG signals are extracted from EEG data using the microstate-based method, which further subdivides the components of the EEG, resulting in more abundant neural information. Then, the traditional EEG methods such as the full-signal power spectra analysis are employed to analyze the microstate EEG signals. As a result, this approach may be more likely to detect differences between different conditions or among different subjects compared to the traditional EEG techniques directly used for analyzing the EEG signals. Therefore, by extracting different types of microstate EEG signals via microstate sequences, the study conducted a comparative analysis of individuals with different levels of FoH from a new perspective of microstate power spectra.

We observed excessive relative delta power activity in the s-FoH group for microstates B, C and F compared to the no-FoH and m-FoH groups, mainly located at the frontal and frontal-central areas. Furthermore, these brain regions belong to the frontal cortex and the dorsolateral prefrontal cortex in terms of anatomical structure, which plays an essential role in emotional processing and regulation of affective states (Whittle et al., 2006; Price and Drevets, 2012; Dolan, 2002). Taken together, greater relative delta power of the study probably signified that the brain tended to be abnormal. To some extent, it was highly likely that the s-FoH group had experienced brain dysfunction for the regulation of fear of heights observed during the virtual high-altitude scenario, leading to the emergence of aberrant behaviors. In addition, we also found that s-FoH group showed decreased frontal and frontal-central hypoactivity of alpha band than no-FoH and m-FoH groups for microstates D, F. An earlier research has confirmed that alpha recorded on the scalp was inversely related to the underlying cortical activity (Allen et al., 2004). That is to say, a decreased alpha band power indicates greater cortical hyperexcitabilityA related study based on rs-fMRI revealed that compared to normal controls, increased regional homogeneity (ReHo) in the right fusiform gyrus and decreased ReHo in the bilateral superior frontal gyrus were observed in patients with FoH (Hang et al., 2022). Thus, these observations suggested that virtual high-altitude could have the same influence on delta power of multiple microstates but the opposite impact on alpha band. Moreover, neural oscillatory activities of individuals with severe fear of heights were the most severely affected by the virtual high-altitude.

In the present study, we observed that the power ratio theta/alpha in the s-FoH group was significantly higher than the no-FoH group in frontal-central (i.e. FC2 and FC5) areas for multiple microstates (B, C, D and F), and than the m-FoH group in C3 region for only microstate D. Our results also indicated remarkably greater theta/alpha in the central brain region in m-FoH group for microstate A when compared to no-FoH group. However, theta/alpha did not show a consistent monotonic increase across all microstates, channels, and group comparisons.

Additionally, consistent with previous research demonstrating correlations between brain states and neural oscillation patterns, our study also revealed that the severity of fear of heights had a significant negative relationship with relative alpha power for microstates B, C and D, but positively correlated with relative delta power for microstates B and C, and theta/alpha for microstates A, B, D and F, suggesting the power characteristics of microstate EEG signals could potentially assist the diagnosis and guide therapeutic intervention of fear of heights.

### 4.2 Abnormal topological patterns of functional brain networks for microstate F in people with fear of heights under the exposure of virtual high-altitude

Since all microstate time windows of the same microstate correspond to the characteristics of a specific brain network, microstate-window-based EEG signals used to construct brain networks offer new insights into dynamic brain function activity (Chu et al., 2023). In the current study, we established microstate functional brain network based on microstate-window signals by evaluating the synchronization of any pair of EEG channels using wPLI, and then compared the topological properties of microstate brain network in people with different severity of FoH under the virtual high-altitude scene.

The results showed that the normalized path length, normalized clustering coefficient, normalized local efficiency, and normalized global efficiency among different FoH groups only had significant differences under a few (or a single) sparsity values. It was indicated that different levels of sparsity had different effects on the network topology, which resulted in completely different outcomes. However, clustering coefficient and local efficiency of the brain network for microstate F exhibited significant differences between individuals with different levels of FoH across a wide sparsity range, reflecting that the results were stable under different sparsity values. Therefore, when performed the analysis of network topological properties, it was recommended to choose a relatively wide range of sparsity to avoid the randomness or subjectivity of the results caused by a single sparsity value.

Our results showed that clustering coefficient and local efficiency were abnormally increased in both m-FoH and s-FoH groups relative to no-FoH group, which mainly involved microstate F for almost the whole selected sparsity. The clustering coefficient is a measure of functional segregation (i.e. local structure and regional connectivity) of the network by evaluating the number of clustered connectivity around individual nodes (Rubinov and Sporns, 2010). The local efficiency plays a similar role to the clustering coefficient, which describes the efficiency of information processing in a local cliquiness (it can be understood as a local brain region in the brain network) of a network (Latora and Marchiori, 2001). These network alterations in people with FoH may tentatively point toward altered local brain communication during the virtual high-altitude exposure. Alternative interpretations should be considered. For instance, such changed network patterns could reflect less flexible network configuration. They might reflect compensatory recruitment of additional neural resources to maintain emotional regulation under the virtual high-altitude-related stress. They could also represent adaptive reorganization in response to acute threat, or state-dependent changes that are specific to the experimental context rather than stable trait-like alterations. Previous studies have proven the small-world topology in large-scale functional network in the human brain (Tian et al., 2011; Watts and Strogatz, 1998), which is characterized by high local clustering than the random network and short path length equivalent to the comparable random network. This optimal network configuration reflects the two fundamental organizational principles in the brain: functional segregation and functional integration. However, in this study, the functional brain networks of microstate F in the three groups were found to have approximately the same clustering coefficient and characteristic path length as the matched random networks (i.e.λ≈1andγ≈1), indicating a shift randomization of their brain networks. One reasonable explanation for the results of the study was that the external environment, such as the virtual high-altitude in this study, might has altered the parameters of microstate functional brain network. Interestingly, another EEG study reported that the brain networks of both healthy controls and patients with depression exhibited the characteristics of a regular network during the process of emotional picture stimuli (Li et al., 2015), which supported our explanation to some extent.

Although these three groups showed the exponentially truncated power law of node degree distribution for microstate brain network F, the estimated power law exponent (α) and exponential cutoff (kc) were different among them (no-FoH: α = −2.639, kc = 0.211; m-FoH: α = −8.462, kc = 0.115; s-FoH: α = −11.830, kc = 0.090). It can be seen that the degree distributions of the m-FoH and s-FoH groups were comparable to each other, but differed from that of the no-FoH group. To be specific, both the m-FoH and s-FoH groups had larger absolute α than the no-FoH group on the one hand, suggesting the narrower degree distribution (i.e., the probability of the huge hubs was decreased in brain network in people with fear of heights); while the reduced kc was observed in the m-FoH and s-FoH groups on the other hand, reflecting the transition from the scaling regimen to the exponential fall-off occurs at a lower degree. The results demonstrated that the alterations in the microstate brain network F of FoH groups observed during virtual high-altitude exposure were more profound compared to the no-FoH group.

The fundamental organization of a network is considered to be closely linked to its topological and dynamical properties (Bullmore and Bassett, 2011), suggesting that altered network topology may reflect the deterioration of the network performance such as robustness. In order to verify this hypothesis, the study investigated the robustness of the network to damage in different FoH groups through two ways: random deletion of nodes as well as the edges connected to them and gradually targeted attack on the highest-degree nodes in the network. The results of the study revealed that m-FoH and s-FoH groups had extremely lower network robustness (reduced relative size of the largest cluster and global efficiency) for microstate F than no-FoH group whether the attack was random or targeted, which supported our hypothesis. Meanwhile, the above analysis indicated that the brain networks of the three groups have become randomized during the exposure of virtual high-altitude scenario. In addition, we also found the average small-worldness of microstate brain network F for the no-FoH, m-FoH and s-FoH groups were 0.97, 0.98 and 1.00 respectively, demonstrating that compared to the acrophobic FoH groups, the randomization degree of brain network was relatively more higher in the no-FoH group. The finding was in accordance with a previous fMRI study in which the randomized brain network in depression patients showed greater robustness than healthy subjects during the random failures or targeted attacks (Zhang et al., 2018). In essence, enhanced robustness for the randomization network is closely associated with the decreased hub nodes with greater degree. The proposition was favored by our result indicating that the number of hubs in microstate brain network F in no-FoH group was less than that in m-FoH and s-FoH groups. Hence, we speculated that the reduction of the hub nodes may lead to the functional brain network closer to a randomized configuration. It seems to be a potential protective mechanism of the randomization brain, which assures the global integrity of the brain network in individuals without fear of heights that will not be disintegrated sharply when the nodes and links are attacked. In other words, for the m-FoH and s-FoH groups, the reduced network robustness might signify that its role significantly weakens in combating the loss of network functions due to fear induced by virtual high-altitude. However, we cannot rule out the possibility that the observed network randomization may stem from general neural effects of immersive VR stimulation, instead of reflecting the regulatory processing of virtual high-altitude-induced fear. Future work incorporating a non-high-altitude VR control condition is needed to separate task-evoked network alterations from individual-specific adaptive brain responses. Together, these findings regarding the microstate brain network analysis in individuals with different severity of FoH offered implications for our understanding of the association between the topological structure of brain and neuropathological state of FoH.

Besides, compared with the no-FoH group, the s-FoH group exhibited weaker functional connectivity strength between the right-upper and left-lower brain regions, between the right-upper and midline brain regions, as well as within the midline brain regions for microstate A. The right-upper brain region encompasses the right frontal areas, which are involved in emotional processing, particularly for negative emotions such as fear (Wyczesany et al., 2018; Gainotti, 2024; TenHouten, 2026). Furthermore, a virtual reality-EEG-ECG study has demonstrated that the midline brain region is implicated in threat perception (Lu et al., 2023). Taken together, the reduced functional connectivity for microstate A in the s-FoH group mainly involved right frontal and midline brain regions, which are closely associated with emotional processing and threat perception, reflecting altered network interactions in individuals with severe fear of heights.

In summary, the above results demonstrated that the significant differences of power features of microstate EEG between individuals with different levels of FoH were associated with multiple types of microstate classes including the microstate F, but the topological properties of microstate brain networks were mainly closely related to the microstate F. Combined with the results of our previous research (Teng et al., 2024), that is, the differences in microstate basic characteristics among three FoH groups were also closely associated with the microstate F, which was consistent with the results of the study to a certain extent, indicating that the microstate F was likely to play an important role in the regulation of fear under the virtual high-altitude condition. Given the cross-sectional nature of the present study, the observed microstate EEG power and network features may identify potentially relevant electrophysiological features for virtual high-altitude-induced fear, but these features serving as diagnostic biomarkers to assist in early diagnosis, monitor disease progression, or evaluate the treatment efficacy are not yet validated, which should be regarded as possible directions for future confirmation in independent samples, broader populations, and longitudinal or intervention studies.

### 4.3 Achieved statistical power

Regarding statistical power, the present study did not employ an a priori sample-size calculation. The sample (*N* = 69) was derived from our previous study and was therefore based on convenience. To evaluate the sensitivity of our main comparisons, we conducted *post-hoc* power analyses for the one-way ANOVA. The observed effect sizes mainly ranged from η^2^ = 0.09 to 0.15 (corresponding to effect size *f* ≈ 0.31–0.42), and the achieved power ranged from 0.62 to 0.87. For the comparisons with η^2^ ≥ 0.13, power exceeded the conventional 0.80 threshold, suggesting that these analyses were adequately sensitive to detect medium-to-large effects. In contrast, for comparisons with η^2^ = 0.09–0.12, the achieved power was 0.62–0.77, which is below the recommended 0.80 level. This suggests that some of the main comparisons may have been underpowered to detect small-to-moderate effects.

### 4.4 Methodological issues

In order to explore the inherent spectral characteristics of different microstates, the study attempted to use an analysis method based on the concatenation of the segments of microstate time windows, aiming to conduct the power spectrum analysis on the transient neural oscillation segments that occurred at different time points and belonged to the same type of microstate, which reflect analogous brain activation pattern. Therefore, the concatenation is physiological reasonable. Considering that the duration of a single segment was too short and direct Fourier transformation would result in insufficient frequency resolution, the study constructed a longer synthetic time series by concatenating all the same type of microstate segments, and then calculated its power spectral density. The cumulative signal duration available for each microstate and each subject is presented in Supplementary Table 3. The results of the one-way ANOVA analysis showed that there were no significant differences in the length of EEG signals for microstates A, B, C and D among three groups. The Kruskal–Wallis one-way ANOVA test for multiple independent samples revealed a significant difference in the data length of microstate F between the groups (*H* = 12.69, *p* = 0.002; the mean data length for the no-FoH, m-FoH and s-FoH groups were 23.61 ± 7.37, 16.35 ± 6.33 and 17.33 ± 6.36, respectively). Specifically, the FoH group had significantly lower data length than the no-FoH group (m-FoH vs. no-FoH: H = 21.01, *pcorr* = 0.001; s-FoH vs. no-FoH: H = 15.28, *pcorr* = 0.036). Although the microstate data for each individual can be used for power spectrum analysis, we acknowledge that inter-individual variability in the duration and coverage of each microstate leads to differences in cumulative signal duration across microstates during EEG microstate analysis. Since the power spectrum estimation is effected by the signal duration, future research should collect longer data to minimize the deviation in the power spectrum estimation as much as possible. On the other hand, it must be pointed out that this method of directly concatenating microstate segments artificially introduces discontinuities between adjacent segments, potentially causing spectral leakage during power spectrum analysis. This factor should also be taken into consideration when interpreting the power spectrum findings.

It is thought that brain function activity does not proceed continuously and smoothly, but is concatenated by a series of quasi-stable neural activity states, known as microstates (Lehmann and Koenig, 1997). Based on this assumption, these microstates last for approximately several tens of milliseconds and are regarded as “quantized” processing units that carry out instantaneous thinking or cognition. During this brief but stable time window, the sub-processes distributed across various brain regions will work collaboratively to form a temporary functional network, jointly completing a specific information processing task (Lehmann et al., 2006). Therefore, calculating the functional connections within a single microstate window is an attempt to capture the dynamic network configuration corresponding to this specific “mental moment.” Accordingly, the present study extracted all EEG segments corresponding to the same class of microstate for each subject, and these segments were then used to compute the average FC matrix and construct brain network. This microstate-window-based FC approach preserves the uniqueness of functional brain network associated with the same microstate within each time window, which is help for facilitating the identification of brain network pattern that only occurs in a specific microstate. Nevertheless, the analytical approach still has several limitations. First, although almost each microstate contained an adequate number (more than 30) of valid time windows for each subject, which guaranteed favorable stability and signal-to-noise ratio for the estimated FC, the number of microstate windows varied across participants. Second, time windows of the same microstate class reflect similar global brain activity, while the inherently short duration of individual microstate window inevitably constrains the statistical power of FC calculation and subsequent group-level statistical analysis. To overcome these shortcomings, some improvement methods including collecting more EEG data, setting a relatively longer minimum smoothing segment duration during the back-fitting procedure of microstate analysis and the microstate segment supplementation strategy can be adopted in future studies to balance the number and cumulative duration of valid microstate windows for each subject, and eliminate the confounding effects of uneven data volume on inter-group comparisons.

### 4.5 Limitations

Some limitations should be noted in the study. Firstly, the sample size of the study was relatively small and only male subjects in their 20s were included. The exclusive recruitment of young male participants limits the generalizability of our results. Gender differences in autonomic response, emotional regulation and EEG dynamics may lead to distinct neural manifestations of fear induced by heights in the female population compared to the male population. In addition, age-related brain maturation and functional reorganization may further modulate microstate dynamics and network topological properties in older adults. So, the current brain electrophysiological characteristics across different FoH should only be reliably inferred for young male groups, and a larger group including both genders and people with different ages in future work is needed to be recruited to verify the generalizability of the findings in the study. Secondly, the study did not collect the trait anxiety inventory of each subject. Trait anxiety is a well-established moderator of fear responses and brain activity. Previous studies have reported that individuals with high trait anxiety generally exhibited excessive fear responses (Ming et al., 2025), a relatively lower frontal theta/alpha oscillatory balance (Wang et al., 2013), and altered brain network configurations (Zhang et al., 2024). Since trait anxiety was not measured and excluded as a covariant, the observed differences in microstate EEG characteristics in spectral ratios and network topology between the groups cannot be exclusively attributed to fear of height itself. It remains possible that a baseline anxiety predisposition partially overlaps with, or contributes to, the reported electrophysiological brain alterations. Although the no-FoH, m-FoH and s-FoH groups were matched for basic demographic variables and recruited from a homogeneous young male population, subtle unmeasured differences in trait anxiety may still confound group-level neural patterns. Future work should incorporate the trait anxiety inventories of all subjects and adopt the covariance analysis to eliminate anxiety-related confounds, determining whether the group differences observed are due to a pre-existing trait anxiety or a state effect of VR-induced fear. Thirdly, the brain network construction in the study was based on the scalp EEG level rather than the cortical source level, which might introduce non-neuronal activities due to the effect of volume conduct, limiting our interpretation of the results. Although the wPLI method used in the study weakens the volume conduction influence (Vinck et al., 2011), the analysis from the source space could provide more anatomically specific localization of network nodes, which is helpful for us to further deeply understand the pathology of fear of heights. Future investigations combining individual MRI and functional network analysis at sensor level [e.g., using exact low resolution electromagnetic tomography (eLORETA)] are needed to validate and extend our present findings. Fourthly, the microstate-based technique have been employed for the analysis of EEG signals to investigate brain function activity in the study, but it is unknown whether the traditional EEG methods such as full-signal power spectra directly applied to EEG signals can yield different results. Therefore, the future research should conduct a comparative analysis of these two types of methods to determine whether the microstate-based approach has a greater advantage over the traditional EEG methods. Furthermore, we did not record the resting-state EEG data as a baseline, so the observed group differences cannot be conclusively attributed to changes induced by virtual high-altitude exposure. Future research should collect the resting-state EEG signals before virtual high-altitude exposure to control for the baseline differences of EEG characteristics between the groups. In addition, another limitation is that a 2-20 Hz band-pass filter used in the study inherited from prior microstate analysis, which excludes high beta and gamma frequencies associated with emotional processing and cognitive control. However, this choice was methodologically dictated by the microstate analysis framework, and future studies containing high frequencies analyses may complement our findings.

## 5 Conclusions

In conclusion, the study investigated brain function activity in people with different severity of fear of heights during the exposure of virtual high-altitude based on the analyses of power spectrum and functional brain network for microstate EEG signals. The results revealed significant alterations of both m-FoH and s-FoH groups in the characteristics of microstate EEG oscillatory power and microstate brain network topology mainly closely associated with the microstate F contributing to our understanding of the neural mechanism of fear of heights, FoH needs to be further verified in the future.

## Data Availability

The original contributions presented in the study are included in the article/Supplementary material, further inquiries can be directed to the corresponding authors.
