# Supplementary material for: A study on the effects of virtual high-altitude exposure on brain function activity in people with different severity of fear of heights
Source: Front Psychol. 2026 Oct 7;17:1934322. doi: 10.3389/fpsyg.2026.1934322 (PMC13683205; doi:10.3389/fpsyg.2026.1934322)
Supplement: Supplementary file 1 [file Table_1.docx]

**Supplemental materials**

**FIGURE S1 |** Distributions of brain topographic maps for average relative power of basic EEG rhythms of different FoH groups for microstate A. Relative (A) delta, (B) theta, (C) alpha, and (D) beta power. Each row of each subgraph represents the average relative power results of of the no-FoH, m-FoH and s-FoH groups based on EEG signals of microstate A. FoH: fear of heights; the no-FoH, m-FoH and s-FoH denote no, mild or moderate and severe fear of heights, respectively.

**FIGURE S2 |** Distributions of brain topographic maps for average relative power of basic EEG rhythms of different FoH groups for microstate B. Relative (A) delta, (B) theta, (C) alpha, and (D) beta power. Each row of each subgraph represents the average relative power results of of the no-FoH, m-FoH and s-FoH groups based on EEG signals of microstate B. For the explanation of the abbreviations in the figure, please refer to **FIGURE S1**.

**FIGURE S3 |** Distributions of brain topographic maps for average relative power of basic EEG rhythms of different FoH groups for cicrostate C. Relative (A) delta, (B) theta, (C) alpha, and (D) beta power. Each row of each subgraph represents the average relative power results of of the no-FoH, m-FoH and s-FoH groups based on EEG signals of microstate C. For the explanation of the abbreviations in the figure, please refer to **FIGURE S1**.

**FIGURE S4 |** Distributions of brain topographic maps for average relative power of basic EEG rhythms of different FoH groups for microstate D. Relative (A) delta, (B) theta, (C) alpha, and (D) beta power. Each row of each subgraph represents the average relative power results of of the no-FoH, m-FoH and s-FoH groups based on EEG signals of microstate D. For the explanation of the abbreviations in the figure, please refer to **FIGURE S1**.

**FIGURE S5 |** Distributions of brain topographic maps for average power ratios of EEG rhythms of different FoH groups for microstate A. (A) theta/alpha, (B) theta/beta, and (C) alpha/beta. Each row of each subgraph represents the average power ratio results of the no-FoH, m-FoH and s-FoH groups based on EEG signals of microstate A. For the explanation of the abbreviations in the figure, please refer to **FIGURE S1**.

**FIGURE S6 |** Distributions of brain topographic maps for average power ratios of EEG rhythms of different FoH groups for microstate B. (A) theta/alpha, (B) theta/beta, and (C) alpha/beta. Each row of each subgraph represents the average power ratio results of the no-FoH, m-FoH and s-FoH groups based on EEG signals of microstate B. For the explanation of the abbreviations in the figure, please refer to **FIGURE S1**.

**FIGURE S7 |** Distributions of brain topographic maps for average power ratios of EEG rhythms of different FoH groups for microstate C. (A) theta/alpha, (B) theta/beta, and (C) alpha/beta. Each row of each subgraph represents the average power ratio results of the no-FoH, m-FoH and s-FoH groups based on EEG signals of microstate C. For the explanation of the abbreviations in the figure, please refer to **FIGURE S1**.

**FIGURE S8 |** Distributions of brain topographic maps for average power ratios of EEG rhythms of different FoH groups for microstate F. (A) theta/alpha, (B) theta/beta, and (C) alpha/beta. Each row of each subgraph represents the average power ratio results of the no-FoH, m-FoH and s-FoH groups based on EEG signals of microstate F. For the explanation of the abbreviations in the figure, please refer to **FIGURE S1**.

**FIGURE S9 |** Comparison results of brain network properties of microstate A as a function of sparsity for different FoH groups. The network metrics (a) *L*, (b) *C*, (c) *E_glo_*, (d) *E_loc_*, (e) *λ*, (f) *γ*, (g) normalized *E_glo_* and (h) normalized *E_loc_* denote characteristic path length, clustering coefficient, global efficiency, local efficiency, normalized characteristic path length, normalized clustering coefficient, normalized global efficiency and normalized local efficiency, respectively. Shaded areas around the mean values represent the standard errors. The ~~red asterisks *~~ squares □ indicate significant differences between the no-FoH and m-FoH groups, the ~~green asterisks *~~ circles ○ for significant differences of the no-FoH and s-FoH groups, and the ~~blue asterisks *~~ triangles △ for significant differences of the m-FoH and s-FoH groups (*p* < 0.05, Bonferroni correction). FoH: fear of heights; no-FoH: no fear of heights; m-FoH: mild or moderate fear of heights; s-FoH: severe fear of heights.

**FIGURE S10 |** Comparison results of brain network properties of microstate C as a function of sparsity for different FoH groups. The network metrics (a) *L*, (b) *C*, (c) *E_glo_*, (d) *E_loc_*, (e) *λ*, (f) *γ*, (g) normalized *E_glo_* and (h) normalized *E_loc_* denote characteristic path length, clustering coefficient, global efficiency, local efficiency, normalized characteristic path length, normalized clustering coefficient, normalized global efficiency and normalized local efficiency, respectively. For the explanation of the abbreviations in the figure, please refer to **FIGURE S9**.

**FIGURE S11 |** Comparison results of brain network properties of microstate D as a function of sparsity for different FoH groups. The network metrics (a) *L*, (b) *C*, (c) *E_glo_*, (d) *E_loc_*, (e) *λ*, (f) *γ*, (g) normalized *E_glo_* and (h) normalized *E_loc_* denote characteristic path length, clustering coefficient, global efficiency, local efficiency, normalized characteristic path length, normalized clustering coefficient, normalized global efficiency and normalized local efficiency, respectively. For the explanation of the abbreviations in the figure, please refer to **FIGURE S9**.

**FIGURE S12 |** The degree distributions of functional brain networks of microstate B for different FoH groups. (A) The histograms of regional degree *k* distributions for different FoH groups, (B) the fitted curves of the logarithm of degree log(*k*) versus the logarithm of cumulative probability of degree under three degree distribution forms. The red circle **○** represents the observation data of the no-FoH group, the green circle ○ for the observation data of the m-FoH group, and the blue circle ○ for the observation data of the s-FoH group. The sign **+** indicates the fit of an exponential (*P*(*k*) ~ *e^ak^*), the dashed line -- for the fit of a power law (*P*(*k*) ~ *k^α^*^-1^), and the dotted line -. for the fit of an exponentially truncated power law (*P*(*k*) ~ *k^α^*^-1^e*^k^*^/^*^k^*^c^). Re denotes the coefficient of determination *R*^2^ for the exponential fit, Rp for the power law fit, and Retp for the exponentially truncated power-law fit. FoH: fear of heights; no-FoH: no fear of heights; m-FoH: mild or moderate fear of heights; s-FoH: severe fear of heights. According to the estimated *R*^2^ values, the degree distributions of all groups were best fitted by the power law (no-FoH: *α* = 8.629; m-FoH: *α* = 6.367; s-FoH: *α* = 5.582).

**FIGURE S13 |** The degree distributions of functional brain networks of microstate C for different FoH groups. (A) The histograms of regional degree *k* distributions for different FoH groups, (B) the fitted curves of the logarithm of degree log(*k*) versus the logarithm of cumulative probability of degree under three degree distribution forms. For the explanation of the abbreviations in the figure, please refer to **FIGURE S12**. According to the estimated *R*^2^ values, the degree distributions of all groups were best fitted by the exponentially truncated power law (no-FoH: *α* = -5.214, *kc* = 0.143; m-FoH: *α* = -8.471, *kc* = 0.123; s-FoH: *α* = 2.456, *kc* = 0.586).

**FIGURE S14 |** The degree distributions of functional brain networks of microstate C for different FoH groups. (A) The histograms of regional degree *k* distributions for different FoH groups, (B) the fitted curves of the logarithm of degree log(*k*) versus the logarithm of cumulative probability of degree under three degree distribution forms. For the explanation of the abbreviations in the figure, please refer to **FIGURE S12**. According to the estimated *R*^2^ values, the degree distributions of all groups were best fitted by the exponentially truncated power law (no-FoH: *α* = -2.639, *kc* = 0.211; m-FoH: *α* = -8.462, *kc* = 0.115; s-FoH: *α* = -11.830, *kc* = 0.090).

**TABLE S1 |** Number of microstate windows used for wPLI estimation for each subject.

| Group | NO. | Number of microstate windows for microstates | | | | |
| --- | --- | --- | --- | --- | --- | --- |
|  |  | A | B | C | D | F |
| no-FoH | no-FoH-1 | 114 | 114 | 54 | 114 | 156 |
|  | no-FoH-2 | 71 | 57 | 209 | 71 | 115 |
|  | no-FoH-3 | 127 | 118 | 48 | 127 | 190 |
|  | no-FoH-4 | 99 | 111 | 172 | 99 | 90 |
|  | no-FoH-5 | 130 | 54 | 165 | 130 | 115 |
|  | no-FoH-6 | 31 | 66 | 176 | 31 | 151 |
|  | no-FoH-7 | 156 | 169 | 92 | 156 | 87 |
|  | no-FoH-8 | 158 | 120 | 115 | 158 | 75 |
|  | no-FoH-9 | 127 | 80 | 90 | 127 | 130 |
|  | no-FoH-10 | 63 | 132 | 99 | 63 | 200 |
|  | no-FoH-11 | 179 | 131 | 112 | 179 | 88 |
|  | no-FoH-12 | 69 | 97 | 219 | 69 | 93 |
|  | no-FoH-13 | 113 | 206 | 107 | 113 | 71 |
|  | no-FoH-14 | 139 | 135 | 127 | 139 | 96 |
|  | no-FoH-15 | 123 | 102 | 211 | 123 | 96 |
|  | no-FoH-16 | 59 | 20 | 212 | 59 | 167 |
|  | no-FoH-17 | 45 | 83 | 155 | 45 | 156 |
|  | no-FoH-18 | 60 | 78 | 171 | 60 | 159 |
|  | no-FoH-19 | 87 | 205 | 74 | 87 | 71 |
|  | no-FoH-20 | 54 | 144 | 260 | 54 | 79 |
|  | Mean | 100.2 | 111.1 | 143.4 | 100.2 | 119.3 |
|  | Std | 42.3 | 47.8 | 60.2 | 42.3 | 41.1 |
| m-FoH | m-FoH-1 | 82 | 125 | 145 | 82 | 83 |
|  | m-FoH-2 | 64 | 80 | 209 | 64 | 37 |
|  | m-FoH-3 | 73 | 131 | 224 | 73 | 33 |
|  | m-FoH-4 | 78 | 119 | 31 | 78 | 32 |
|  | m-FoH-5 | 29 | 78 | 184 | 29 | 77 |
|  | m-FoH-6 | 93 | 80 | 251 | 93 | 53 |
|  | m-FoH-7 | 112 | 83 | 206 | 112 | 72 |
|  | m-FoH-8 | 71 | 126 | 178 | 71 | 64 |
|  | m-FoH-9 | 211 | 193 | 15 | 211 | 140 |
|  | m-FoH-10 | 72 | 153 | 74 | 72 | 67 |
|  | m-FoH-11 | 34 | 174 | 158 | 34 | 62 |
|  | m-FoH-12 | 160 | 103 | 197 | 160 | 43 |
|  | m-FoH-13 | 167 | 118 | 199 | 167 | 49 |
|  | m-FoH-14 | 144 | 246 | 37 | 144 | 94 |
|  | m-FoH-15 | 157 | 142 | 24 | 157 | 136 |
|  | m-FoH-16 | 114 | 205 | 64 | 114 | 74 |
|  | m-FoH-17 | 62 | 90 | 252 | 62 | 41 |
|  | m-FoH-18 | 105 | 39 | 210 | 105 | 94 |
|  | m-FoH-19 | 65 | 101 | 57 | 65 | 143 |
|  | m-FoH-20 | 85 | 161 | 147 | 85 | 139 |
|  | m-FoH-21 | 10 | 72 | 200 | 10 | 113 |
|  | m-FoH-22 | 172 | 138 | 64 | 172 | 70 |
|  | m-FoH-23 | 44 | 50 | 250 | 44 | 46 |
|  | m-FoH-24 | 98 | 63 | 74 | 98 | 72 |
|  | m-FoH-25 | 86 | 73 | 157 | 86 | 155 |
|  | Mean | 95.5 | 117.7 | 144.3 | 95.5 | 79.6 |
|  | Std | 49.5 | 50.9 | 79.2 | 49.5 | 37.9 |
| s-FoH | s-FoH-1 | 67 | 109 | 94 | 67 | 97 |
|  | s-FoH-2 | 85 | 91 | 149 | 85 | 116 |
|  | s-FoH-3 | 170 | 180 | 93 | 170 | 67 |
|  | s-FoH-4 | 128 | 175 | 103 | 128 | 76 |
|  | s-FoH-5 | 177 | 122 | 197 | 177 | 48 |
|  | s-FoH-6 | 110 | 140 | 126 | 110 | 104 |
|  | s-FoH-7 | 162 | 156 | 44 | 162 | 146 |
|  | s-FoH-8 | 80 | 111 | 198 | 80 | 13 |
|  | s-FoH-9 | 88 | 160 | 132 | 88 | 42 |
|  | s-FoH-10 | 212 | 110 | 168 | 212 | 57 |
|  | s-FoH-11 | 202 | 112 | 31 | 202 | 111 |
|  | s-FoH-12 | 110 | 117 | 129 | 110 | 152 |
|  | s-FoH-13 | 80 | 120 | 121 | 80 | 95 |
|  | s-FoH-14 | 62 | 125 | 193 | 62 | 87 |
|  | s-FoH-15 | 215 | 104 | 68 | 215 | 86 |
|  | s-FoH-16 | 170 | 165 | 75 | 170 | 84 |
|  | s-FoH-17 | 117 | 105 | 171 | 117 | 40 |
|  | s-FoH-18 | 66 | 67 | 243 | 66 | 29 |
|  | s-FoH-19 | 43 | 74 | 166 | 43 | 126 |
|  | s-FoH-20 | 38 | 89 | 198 | 38 | 88 |
|  | s-FoH-21 | 96 | 94 | 186 | 96 | 54 |
|  | s-FoH-22 | 30 | 63 | 250 | 30 | 64 |
|  | s-FoH-23 | 29 | 21 | 237 | 29 | 70 |
|  | s-FoH-24 | 140 | 71 | 115 | 140 | 134 |
|  | Mean | 111.5 | 111.7 | 145.3 | 111.5 | 82.8 |
|  | Std | 58.0 | 38.5 | 61.3 | 58.0 | 36.6 |

wPLI: weight phase lag index; FoH: fear of heights; The no-FoH, m-FoH and s-FoH denote no, mild or moderate and severe fear of heights, respectively.

**TABLE S2 |** Cumulative signal duration of microstate windows used for wPLI estimation for each subject (unit: s).

| Group | NO. | Cumulative signal duration of microstate windows for microstates | | | | |
| --- | --- | --- | --- | --- | --- | --- |
|  |  | A | B | C | D | F |
| no-FoH | no-FoH-1 | 12.16 | 11.92 | 5.42 | 12.16 | 17.43 |
|  | no-FoH-2 | 6.90 | 5.36 | 23.86 | 6.90 | 12.07 |
|  | no-FoH-3 | 13.04 | 11.50 | 4.57 | 13.04 | 20.65 |
|  | no-FoH-4 | 10.14 | 11.60 | 18.44 | 10.14 | 8.63 |
|  | no-FoH-5 | 13.35 | 5.38 | 17.72 | 13.35 | 11.68 |
|  | no-FoH-6 | 2.66 | 6.24 | 19.04 | 2.66 | 15.96 |
|  | no-FoH-7 | 17.03 | 18.34 | 9.28 | 17.03 | 8.59 |
|  | no-FoH-8 | 17.69 | 12.48 | 11.49 | 17.69 | 7.57 |
|  | no-FoH-9 | 12.85 | 8.35 | 9.13 | 12.85 | 14.05 |
|  | no-FoH-10 | 6.68 | 13.84 | 10.16 | 6.68 | 21.60 |
|  | no-FoH-11 | 20.21 | 14.50 | 11.64 | 20.21 | 8.68 |
|  | no-FoH-12 | 6.57 | 9.53 | 25.12 | 6.57 | 9.21 |
|  | no-FoH-13 | 11.55 | 21.86 | 10.11 | 11.55 | 6.92 |
|  | no-FoH-14 | 14.84 | 13.44 | 13.10 | 14.84 | 9.61 |
|  | no-FoH-15 | 11.96 | 10.08 | 23.37 | 11.96 | 10.23 |
|  | no-FoH-16 | 5.45 | 1.80 | 23.48 | 5.45 | 17.82 |
|  | no-FoH-17 | 4.10 | 8.23 | 17.25 | 4.10 | 17.03 |
|  | no-FoH-18 | 6.23 | 8.22 | 19.03 | 6.23 | 16.77 |
|  | no-FoH-19 | 8.61 | 23.55 | 7.42 | 8.61 | 7.20 |
|  | no-FoH-20 | 5.13 | 14.87 | 28.52 | 5.13 | 8.07 |
|  | Mean | 10.36 | 11.55 | 15.41 | 10.36 | 12.49 |
|  | Std | 4.89 | 5.45 | 7.10 | 4.89 | 4.75 |
| m-FoH | m-FoH-1 | 8.31 | 12.86 | 15.33 | 8.31 | 8.29 |
|  | m-FoH-2 | 6.20 | 7.64 | 23.91 | 6.20 | 3.23 |
|  | m-FoH-3 | 7.22 | 13.11 | 26.37 | 7.22 | 3.05 |
|  | m-FoH-4 | 7.91 | 12.37 | 2.93 | 7.91 | 2.78 |
|  | m-FoH-5 | 2.94 | 7.16 | 20.63 | 2.94 | 7.47 |
|  | m-FoH-6 | 9.21 | 8.16 | 28.93 | 9.21 | 4.73 |
|  | m-FoH-7 | 11.27 | 8.37 | 22.71 | 11.27 | 6.82 |
|  | m-FoH-8 | 7.02 | 12.45 | 18.66 | 7.02 | 6.62 |
|  | m-FoH-9 | 23.35 | 21.54 | 1.36 | 23.35 | 14.37 |
|  | m-FoH-10 | 7.01 | 16.51 | 7.52 | 7.01 | 6.30 |
|  | m-FoH-11 | 3.07 | 19.46 | 16.67 | 3.07 | 6.07 |
|  | m-FoH-12 | 18.31 | 10.72 | 21.66 | 18.31 | 4.52 |
|  | m-FoH-13 | 18.75 | 12.43 | 21.55 | 18.75 | 4.64 |
|  | m-FoH-14 | 15.89 | 29.25 | 3.47 | 15.89 | 9.13 |
|  | m-FoH-15 | 16.37 | 14.73 | 2.36 | 16.37 | 13.96 |
|  | m-FoH-16 | 11.85 | 23.85 | 6.02 | 11.85 | 7.21 |
|  | m-FoH-17 | 6.08 | 9.21 | 31.25 | 6.08 | 3.91 |
|  | m-FoH-18 | 10.85 | 3.68 | 24.08 | 10.85 | 9.38 |
|  | m-FoH-19 | 6.28 | 10.62 | 5.42 | 6.28 | 14.79 |
|  | m-FoH-20 | 8.30 | 16.69 | 14.83 | 8.30 | 14.51 |
|  | m-FoH-21 | 0.82 | 7.43 | 21.86 | 0.82 | 12.05 |
|  | m-FoH-22 | 18.09 | 15.10 | 6.33 | 18.09 | 6.68 |
|  | m-FoH-23 | 4.22 | 4.80 | 28.47 | 4.22 | 4.17 |
|  | m-FoH-24 | 9.65 | 6.24 | 6.90 | 9.65 | 6.68 |
|  | m-FoH-25 | 8.44 | 7.21 | 16.84 | 8.44 | 17.16 |
|  | Mean | 9.90 | 12.46 | 15.84 | 9.90 | 7.94 |
|  | Std | 5.66 | 6.19 | 9.47 | 5.66 | 4.20 |
| s-FoH | s-FoH-1 | 6.59 | 11.40 | 9.68 | 6.59 | 9.98 |
|  | s-FoH-2 | 8.47 | 9.43 | 16.39 | 8.47 | 11.81 |
|  | s-FoH-3 | 19.60 | 21.23 | 9.51 | 19.60 | 6.57 |
|  | s-FoH-4 | 12.59 | 19.61 | 10.13 | 12.59 | 6.80 |
|  | s-FoH-5 | 18.90 | 12.53 | 21.45 | 18.90 | 4.29 |
|  | s-FoH-6 | 11.09 | 14.78 | 12.56 | 11.09 | 10.49 |
|  | s-FoH-7 | 18.62 | 16.24 | 3.91 | 18.62 | 15.14 |
|  | s-FoH-8 | 7.74 | 11.12 | 22.40 | 7.74 | 1.24 |
|  | s-FoH-9 | 8.47 | 16.87 | 14.16 | 8.47 | 3.87 |
|  | s-FoH-10 | 24.09 | 11.55 | 18.45 | 24.09 | 5.88 |
|  | s-FoH-11 | 23.19 | 11.10 | 2.68 | 23.19 | 11.23 |
|  | s-FoH-12 | 11.57 | 11.25 | 13.33 | 11.57 | 17.17 |
|  | s-FoH-13 | 7.68 | 12.15 | 12.61 | 7.68 | 9.89 |
|  | s-FoH-14 | 6.23 | 12.70 | 20.24 | 6.23 | 8.57 |
|  | s-FoH-15 | 23.86 | 10.61 | 6.08 | 23.86 | 8.50 |
|  | s-FoH-16 | 17.80 | 17.48 | 7.39 | 17.80 | 8.71 |
|  | s-FoH-17 | 12.77 | 10.36 | 19.04 | 12.77 | 4.17 |
|  | s-FoH-18 | 6.52 | 6.83 | 27.35 | 6.52 | 2.68 |
|  | s-FoH-19 | 3.95 | 7.21 | 18.21 | 3.95 | 13.50 |
|  | s-FoH-20 | 3.58 | 9.35 | 21.53 | 3.58 | 8.50 |
|  | s-FoH-21 | 9.23 | 9.84 | 20.38 | 9.23 | 5.06 |
|  | s-FoH-22 | 2.75 | 5.64 | 29.16 | 2.75 | 6.05 |
|  | s-FoH-23 | 2.54 | 1.81 | 26.32 | 2.54 | 6.95 |
|  | s-FoH-24 | 15.02 | 7.05 | 11.54 | 15.02 | 13.20 |
|  | Mean | 11.79 | 11.59 | 15.60 | 11.79 | 8.34 |
|  | Std | 6.84 | 4.49 | 7.31 | 6.84 | 4.01 |

wPLI: weight phase lag index; FoH: fear of heights; The no-FoH, m-FoH and s-FoH denote no, mild or moderate and severe fear of heights, respectively.

**TABLE S3 |** Cumulative signal duration used for spectrum estimation for each microstate and each subject (unit: s).

| Group | NO. | Cumulative signal duration for microstates | | | | |
| --- | --- | --- | --- | --- | --- | --- |
|  |  | A | B | C | D | F |
| no-FoH | no-FoH-1 | 12.96 | 30.10 | 24.07 | 22.80 | 30.07 |
|  | no-FoH-2 | 39.95 | 28.20 | 11.61 | 15.24 | 25.00 |
|  | no-FoH-3 | 10.91 | 22.34 | 21.75 | 25.64 | 39.36 |
|  | no-FoH-4 | 34.38 | 27.48 | 21.54 | 18.63 | 17.97 |
|  | no-FoH-5 | 30.74 | 28.22 | 12.51 | 24.79 | 23.73 |
|  | no-FoH-6 | 33.32 | 34.16 | 14.13 | 7.22 | 31.17 |
|  | no-FoH-7 | 21.24 | 21.21 | 31.98 | 27.51 | 18.05 |
|  | no-FoH-8 | 19.56 | 29.29 | 23.96 | 30.51 | 16.68 |
|  | no-FoH-9 | 18.18 | 33.80 | 17.11 | 25.00 | 25.91 |
|  | no-FoH-10 | 19.71 | 23.05 | 25.36 | 16.20 | 35.68 |
|  | no-FoH-11 | 21.71 | 23.16 | 24.45 | 33.81 | 16.87 |
|  | no-FoH-12 | 41.98 | 27.88 | 18.69 | 12.52 | 18.93 |
|  | no-FoH-13 | 20.34 | 25.14 | 36.04 | 23.14 | 15.34 |
|  | no-FoH-14 | 22.66 | 27.44 | 25.04 | 27.45 | 17.41 |
|  | no-FoH-15 | 39.69 | 21.72 | 19.88 | 20.65 | 18.06 |
|  | no-FoH-16 | 39.45 | 32.32 | 5.61 | 12.00 | 30.62 |
|  | no-FoH-17 | 30.35 | 31.90 | 17.59 | 10.88 | 29.28 |
|  | no-FoH-18 | 34.64 | 25.29 | 16.17 | 14.67 | 29.23 |
|  | no-FoH-19 | 15.30 | 30.35 | 40.57 | 17.53 | 16.25 |
|  | no-FoH-20 | 45.42 | 18.70 | 27.42 | 12.00 | 16.47 |
|  | Mean | 27.62 | 27.09 | 21.77 | 19.91 | 23.61 |
|  | Std | 10.57 | 4.40 | 8.36 | 7.31 | 7.37 |
| m-FoH | m-FoH-1 | 27.57 | 35.25 | 23.92 | 15.88 | 17.38 |
|  | m-FoH-2 | 42.59 | 36.96 | 15.47 | 15.14 | 9.83 |
|  | m-FoH-3 | 43.84 | 27.73 | 22.20 | 16.73 | 9.50 |
|  | m-FoH-4 | 9.72 | 60.77 | 23.95 | 17.41 | 8.14 |
|  | m-FoH-5 | 37.48 | 43.92 | 15.01 | 9.00 | 14.59 |
|  | m-FoH-6 | 47.71 | 26.47 | 16.19 | 18.13 | 11.50 |
|  | m-FoH-7 | 42.13 | 25.20 | 16.31 | 21.77 | 14.60 |
|  | m-FoH-8 | 36.42 | 29.89 | 22.96 | 16.01 | 14.73 |
|  | m-FoH-9 | 4.34 | 15.09 | 37.64 | 38.05 | 24.87 |
|  | m-FoH-10 | 17.93 | 43.47 | 29.52 | 15.21 | 13.87 |
|  | m-FoH-11 | 28.96 | 33.06 | 33.62 | 9.88 | 14.47 |
|  | m-FoH-12 | 34.90 | 24.98 | 19.52 | 30.02 | 10.59 |
|  | m-FoH-13 | 36.88 | 15.17 | 24.31 | 32.09 | 11.55 |
|  | m-FoH-14 | 8.94 | 17.21 | 50.20 | 27.50 | 16.14 |
|  | m-FoH-15 | 7.66 | 29.80 | 26.54 | 30.22 | 25.77 |
|  | m-FoH-16 | 13.63 | 28.66 | 39.53 | 21.73 | 16.45 |
|  | m-FoH-17 | 57.79 | 20.55 | 18.47 | 14.09 | 9.10 |
|  | m-FoH-18 | 37.70 | 29.87 | 10.99 | 22.09 | 19.35 |
|  | m-FoH-19 | 13.32 | 43.74 | 21.77 | 14.24 | 26.94 |
|  | m-FoH-20 | 27.77 | 16.79 | 30.53 | 17.49 | 27.42 |
|  | m-FoH-21 | 39.15 | 41.25 | 15.47 | 3.22 | 20.91 |
|  | m-FoH-22 | 12.85 | 33.05 | 27.75 | 30.80 | 15.55 |
|  | m-FoH-23 | 46.56 | 41.61 | 10.29 | 11.00 | 10.55 |
|  | m-FoH-24 | 16.42 | 52.97 | 15.76 | 20.14 | 14.71 |
|  | m-FoH-25 | 28.41 | 27.28 | 16.02 | 18.16 | 30.13 |
|  | Mean | 28.83 | 32.03 | 23.36 | 19.44 | 16.35 |
|  | Std | 14.90 | 11.61 | 9.54 | 8.21 | 6.33 |
| s-FoH | s-FoH-1 | 20.20 | 42.50 | 22.17 | 14.57 | 20.56 |
|  | s-FoH-2 | 29.26 | 31.28 | 18.65 | 17.74 | 23.07 |
|  | s-FoH-3 | 17.14 | 19.50 | 35.63 | 32.84 | 14.89 |
|  | s-FoH-4 | 20.22 | 23.54 | 34.70 | 24.65 | 16.89 |
|  | s-FoH-5 | 40.23 | 14.32 | 23.19 | 31.63 | 10.63 |
|  | s-FoH-6 | 23.71 | 27.81 | 27.29 | 21.16 | 20.03 |
|  | s-FoH-7 | 9.65 | 20.20 | 29.04 | 33.78 | 27.34 |
|  | s-FoH-8 | 38.11 | 38.49 | 21.86 | 17.33 | 4.21 |
|  | s-FoH-9 | 26.28 | 35.93 | 29.30 | 16.01 | 12.48 |
|  | s-FoH-10 | 34.66 | 11.49 | 22.64 | 38.78 | 12.43 |
|  | s-FoH-11 | 7.61 | 28.71 | 21.86 | 39.60 | 22.22 |
|  | s-FoH-12 | 23.86 | 22.10 | 21.91 | 21.84 | 30.29 |
|  | s-FoH-13 | 21.65 | 41.54 | 22.86 | 15.63 | 18.32 |
|  | s-FoH-14 | 34.70 | 31.18 | 22.87 | 14.20 | 17.05 |
|  | s-FoH-15 | 15.47 | 24.88 | 20.38 | 40.07 | 19.19 |
|  | s-FoH-16 | 16.61 | 23.25 | 32.54 | 30.60 | 16.99 |
|  | s-FoH-17 | 32.05 | 33.28 | 21.68 | 22.52 | 10.46 |
|  | s-FoH-18 | 43.65 | 37.71 | 15.75 | 14.84 | 8.05 |
|  | s-FoH-19 | 30.11 | 38.77 | 15.27 | 9.43 | 26.42 |
|  | s-FoH-20 | 35.00 | 38.04 | 18.79 | 10.20 | 17.97 |
|  | s-FoH-21 | 34.75 | 34.21 | 18.91 | 19.82 | 12.31 |
|  | s-FoH-22 | 48.57 | 36.90 | 12.70 | 8.89 | 12.95 |
|  | s-FoH-23 | 44.94 | 44.32 | 6.84 | 7.19 | 16.71 |
|  | s-FoH-24 | 23.74 | 30.30 | 16.15 | 25.32 | 24.49 |
|  | Mean | 28.01 | 30.43 | 22.21 | 22.03 | 17.33 |
|  | Std | 11.01 | 8.98 | 6.79 | 10.08 | 6.36 |

FoH: fear of heights; The no-FoH, m-FoH and s-FoH denote no, mild or moderate and severe fear of heights, respectively.
